# Supplementary material for: Stress-dependent conformational changes of artemin: Effects of heat and oxidant
Source: PLoS One. 2020 Nov 16;15(11):e0242206. doi: 10.1371/journal.pone.0242206 (PMC7668597; doi:10.1371/journal.pone.0242206)
Supplement: S1 Fig — (DOCX) [file pone.0242206.s001.docx]

**S1 Fig. Amino acid sequence of artemin from *A. urmiana* (GenBank accession no: EU380315.1).**

**1 MATEGARNIG QSAPEGKVQM DCPSRHNFDP ECEKAFVEHI HLELASSYHA WSMWAFYARD**

**61 CKAAVGMTRL CEWASHVSAQ RARRMAAYVL TRGGHVDYKE IPAPKKQGWD NFEDAFSHCV**

**121 ANKKRILTSL QSLYQCCQSK DAHCSNFIQT DMMDEVIAWN KFLSDCLSNI HCIGSQGMGP**

**181 WVFDRWLARI VMSKFKHPKI PSLSTSDLES NIPNELFDAE GDMVRAIKKL**
