# Supplementary material for: Stress-dependent conformational changes of artemin: Effects of heat and oxidant
Source: PLoS One. 2020 Nov 16;15(11):e0242206. doi: 10.1371/journal.pone.0242206 (PMC7668597; doi:10.1371/journal.pone.0242206)
Supplement: S1 File — (PDF) [file pone.0242206.s002.pdf]

Data Availability statement

We confirm that the data provided in our Supporting Information as the "minimal data set", reach the conclusions drawn in the manuscript with related metadata and methods, and any additional data required to replicate the reported study findings. In addition, we also provided the original uncropped and unadjusted gel images as “S1_raw_images” file in the revision.

We would like to provide contact information of three involved authours for ensuring data access. The email contacts include:

Zeinab Takalloo (First Author): z.takalu@yahoo.com

S. Shirin Shahangian (Professor Assistant & Project Advisor): shahangian@guilan.ac.ir

Reza H. Sajedi (Professor of Biochemistry & Project Supervisor): sajedi_r@modares.ac.ir

Temperature / Wavelength (nm)	25 °C	30 °C	50 °C	60 °C	70 °C	80 °C
300	227.31	234.144	83.932	121.371	98.339	50.827
300.5	232.1263	238.1556	84.83143	123.983	100.8198	50.57941
301	237.4827	242.7051	86.08921	127.0757	103.3935	50.7877
301.5	243.35	247.6896	87.50562	130.1596	105.9544	51.12396
302	249.7465	253.1808	89.46374	133.4719	108.7341	51.77492
302.5	256.7318	259.3902	91.84893	137.263	112.0154	52.50581
303	263.9063	265.8793	94.37763	141.0187	115.1656	53.44284
303.5	271.1599	272.5914	96.79919	144.7708	118.2621	54.44368
304	278.3497	279.1824	99.27426	148.6661	121.2766	55.3257
304.5	285.3497	285.52	101.4442	152.3113	124.3838	56.26862
305	291.847	291.1209	103.5334	155.7941	127.1808	57.00106
305.5	298.6035	297.0125	105.8055	159.7141	130.106	58.0953
306	305.5703	302.9016	108.0311	163.6635	132.8989	59.26991
306.5	312.5952	308.8032	110.654	167.7103	135.6764	60.89532
307	319.7823	314.9767	113.3823	171.75	138.5747	62.47652
307.5	327.2973	321.8394	116.4241	176.1285	141.6667	64.4161
308	334.6752	329.0587	119.3863	180.4496	144.4764	66.51128
308.5	341.9402	336.6055	122.2141	184.5636	147.1584	68.30524
309	348.8195	344.0976	124.5639	188.2816	149.9216	69.95925
309.5	354.7866	350.7784	126.6846	191.4499	152.256	71.3634
310	360.7598	357.2469	128.8023	194.6231	154.9887	72.7807
310.5	366.7773	363.387	130.7377	197.5893	157.4756	73.85935
311	372.5315	369.211	133.0773	200.6086	159.9483	75.3429
311.5	377.9355	374.0398	135.1854	203.0419	162.2789	76.52374
312	383.7533	378.8687	137.4986	205.9066	164.826	77.94789
312.5	389.7698	383.4539	139.8603	208.9816	167.2469	79.57088
313	395.9972	388.3059	141.9693	211.9611	169.7608	80.97144
313.5	402.2919	393.0556	143.8387	215.0354	172.4115	82.24918
314	408.2451	397.5797	145.6513	218.1466	174.9458	83.50305
314.5	414.4197	402.1153	147.4158	221.4967	178.0224	84.69969
315	421.4642	407.2708	149.1768	225.3278	181.5519	85.82198
315.5	428.2291	413.0263	151.277	229.3631	185.1746	87.30223
316	434.2709	418.45	153.1199	232.5751	188.4971	88.63919
316.5	439.6347	424.0623	154.7	235.8321	191.4399	90.07861
317	444.4902	429.4195	156.069	239.0977	193.5035	91.48964
317.5	449.1851	434.6614	157.0604	241.8873	194.9335	92.81436
318	452.9889	439.3205	157.988	244.284	195.6174	94.15514
318.5	455.9773	443.4697	158.8391	246.3379	195.7843	95.30178
319	458.3501	446.9281	159.5022	248.0228	196.0285	96.32825
319.5	461.5999	450.1087	160.4505	249.963	196.8905	97.25384
320	465.2318	453.8182	161.9453	252.2285	198.5431	98.38122
320.5	469.3088	457.131	163.755	254.2924	200.9099	99.50833

321	473.3245	460.6182	165.5501	256.5333	203.646	100.7647
321.5	477.0752	463.7785	167.3308	258.6752	206.2876	101.8111
322	481.5358	466.7193	168.9997	260.8881	208.867	102.5956
322.5	485.7735	469.174	170.8338	263.0238	210.9936	103.5152
323	489.6966	471.2747	172.5492	265.2016	212.6946	104.3341
323.5	492.7611	473.304	173.9842	266.9491	213.8686	105.1164
324	495.7139	474.9961	175.2826	268.741	214.8485	105.9273
324.5	498.2294	477.0651	176.5453	270.5946	215.8663	107.0307
325	500.68	478.951	177.8878	272.3953	217.0876	108.1884
325.5	502.9286	481.4191	179.0882	274.1864	218.4254	109.3938
326	504.2275	484.1636	179.9357	275.7624	219.5859	110.5006
326.5	505.712	487.0206	180.6468	277.1829	220.3684	111.3154
327	506.8363	489.3584	181.2605	278.1807	220.8849	111.7612
327.5	507.7375	491.029	181.4891	278.8464	220.9109	111.7813
328	508.3056	492.5252	181.4155	279.0428	220.7961	111.81
328.5	508.7153	493.4397	181.0252	278.8247	220.604	111.5215
329	509.0659	494.0679	180.5788	278.0877	220.7517	111.4879
329.5	509.2085	493.6826	180.041	277.2278	221.2388	111.3123
330	509.7074	492.9255	179.6237	276.6163	222.1315	111.1992
330.5	509.8959	492.0648	179.1443	276.371	223.0729	111.2634
331	509.9666	491.6391	179.1432	276.6946	223.8661	111.5631
331.5	509.8018	491.012	179.3852	277.1518	224.5635	111.7294
332	509.2286	489.9619	179.5018	277.6746	224.7574	111.5728
332.5	508.2514	488.7476	179.603	278.2349	224.5161	111.3017
333	506.9655	487.637	179.5148	278.7679	223.8652	110.906
333.5	505.6186	486.5269	179.3961	278.9167	223.1633	110.7579
334	504.4009	485.38	178.9796	278.1983	222.2581	110.5185
334.5	503.4139	483.6647	178.5412	276.961	221.5329	110.2944
335	502.5582	481.3803	177.7928	275.7845	220.82	110.2612
335.5	501.8172	479.5248	177.1909	274.6942	220.2782	110.4957
336	501.2148	477.6226	176.7985	273.2676	219.7532	110.695
336.5	500.2495	475.7905	176.3573	271.7287	219.214	110.6582
337	498.3715	473.3089	175.8475	270.2233	218.5628	110.3547
337.5	496.1134	470.7215	175.2509	269.0204	217.8149	110.0816
338	492.9191	467.4491	174.5475	268.107	216.8063	109.6611
338.5	489.3465	464.5277	173.58	266.7136	215.428	109.0537
339	485.2199	461.1751	172.5739	264.7976	213.5961	108.298
339.5	480.8827	457.1525	171.1959	262.8625	211.1347	107.471
340	476.2389	453.1293	169.526	260.7749	208.6121	106.5355
340.5	471.8956	449.1236	167.858	258.256	205.821	105.6095
341	467.6277	445.6953	165.887	255.4913	203.0562	104.6564
341.5	462.9566	442.5146	163.7799	252.6245	200.5466	103.4851
342	459.0885	439.9973	162.1004	249.9273	198.7687	102.7322
342.5	455.4604	437.2654	160.644	247.8292	197.2988	102.1075
343	452.601	434.9737	159.5089	246.0149	196.4861	101.7793
343.5	449.7636	432.3723	158.5893	243.9851	195.557	101.4085

344	446.8428	429.2387	157.8252	242.2262	194.2604	101.1126
344.5	443.3348	425.2912	156.87	240.4162	192.6558	100.6189
345	439.2561	420.6622	155.8224	238.2525	190.478	99.84123
345.5	434.6332	415.7964	154.3958	235.7584	188.0075	98.65266
346	428.8802	410.5908	152.1498	233.1378	185.2289	96.81668
346.5	423.0855	405.616	149.8196	230.3363	182.7772	94.79848
347	417.3547	400.5021	147.3416	227.665	180.4381	92.77361
347.5	412.389	395.7248	145.2744	225.5118	179.1029	91.18738
348	407.6499	391.0307	143.4765	223.4747	178.2138	89.98222
348.5	403.7459	386.7492	142.2993	221.605	177.5556	89.2527
349	400.1469	382.5717	141.3743	219.6696	176.9534	88.73651
349.5	396.3884	378.4945	140.8673	217.3695	175.8576	88.31829
350	392.1821	374.392	140.5927	214.5888	174.5025	88.07179
350.5	387.4707	369.9969	139.5455	211.6009	172.6375	87.30075
351	382.308	365.5574	138.031	208.0535	170.2539	85.95361
351.5	376.7775	361.3534	136.0084	204.631	167.3539	84.40309
352	371.2996	357.1686	133.9168	201.4246	164.4786	82.833
352.5	365.762	353.2458	131.4073	198.2941	161.5889	81.21113
353	360.6205	349.4384	129.1949	195.3218	158.6703	79.8944
353.5	356.2662	345.6269	126.9442	192.652	156.0172	78.72706
354	352.1041	341.8146	124.7918	189.968	153.3674	77.58989
354.5	347.889	338.1529	123.3803	187.3962	151.2715	77.13221
355	343.537	333.9116	121.8693	184.7114	149.43	76.42771
355.5	339.249	329.367	120.3343	181.645	147.7691	75.56816
356	334.8367	324.6382	118.7956	178.8329	145.9859	74.57784
356.5	330.2752	319.4293	117.3655	176.0959	144.0582	73.32602
357	325.5055	314.3719	115.6921	173.2733	142.0715	71.78697
357.5	320.8457	309.2002	114.3812	170.5601	139.6959	70.44018
358	316.7375	304.3502	112.8321	168.0525	137.0313	69.09084
358.5	312.7886	300.0632	111.0218	165.6403	134.3123	67.77156
359	308.9477	296.3141	109.5294	163.4703	131.9827	67.22654
359.5	304.889	292.6477	107.9269	161.2126	129.9723	66.53185
360	300.813	289.2329	106.2228	158.6829	128.241	65.94146
360.5	296.5347	285.7103	104.5569	156.5287	126.8011	65.54907
361	291.4881	281.745	102.751	154.1663	125.2731	65.04802
361.5	285.8221	277.756	100.8463	151.4359	123.8886	64.2059
362	279.5042	273.4187	99.28026	148.4606	122.107	63.17798
362.5	273.485	268.7871	97.74139	145.3775	119.9385	61.92785
363	267.4924	264.221	96.31276	142.1775	117.7708	60.56355
363.5	261.9493	259.8607	95.02347	138.9233	115.8725	59.55726
364	256.8703	255.4014	93.7981	135.7317	114.3665	58.38952
364.5	252.3115	251.4638	92.64827	132.5753	113.2598	57.2554
365	248.3759	247.6033	91.5177	129.7545	112.4819	56.40161
365.5	244.7893	243.77	89.9898	126.8623	111.5558	55.57944
366	241.1308	239.9457	87.96636	123.7992	110.5512	54.68287
366.5	237.1902	236.0467	85.94814	120.7861	109.07	53.66549

367	233.1406	231.8422	83.83124	117.904	107.1297	52.56881
367.5	228.9367	227.8203	82.20456	115.2705	104.9599	51.44924
368	224.5195	224.0395	80.58039	112.5702	102.4117	50.60098
368.5	220.2964	220.3332	79.31563	110.3306	100.1988	49.8836
369	215.6603	216.7515	78.37146	108.2845	98.17385	49.14157
369.5	211.2583	213.3234	77.92949	106.664	96.46326	48.53634
370	207.073	209.9309	77.57266	105.2	94.70182	47.78603
370.5	203.3205	205.9462	76.81334	103.5866	93.21963	46.97947
371	199.413	202.172	75.6818	101.9025	91.59938	46.07208
371.5	195.7253	198.3055	74.12532	100.0615	90.12327	45.11058
372	192.3798	194.3731	72.84051	98.23317	88.90889	43.8182
372.5	188.9911	190.6123	71.22732	96.15769	87.43518	42.50373
373	185.9182	187.0735	69.70182	94.22617	85.97798	41.24467
373.5	182.6481	183.6813	68.29756	92.41033	84.72504	40.25941
374	179.3135	180.8126	67.09383	90.57888	83.48015	39.66924
374.5	175.8534	178.2299	66.28735	89.19451	82.10805	39.20677
375	172.2777	175.193	65.32677	87.79665	80.78281	38.68405
375.5	168.4083	172.1151	64.14326	86.25605	79.10754	38.27788
376	164.4883	169.3034	62.68924	84.57897	77.19859	37.94505
376.5	160.9142	166.5307	61.531	82.95686	75.45508	37.49741
377	157.664	163.7369	60.23016	81.28463	73.67148	36.83093
377.5	154.7137	160.7403	59.11402	79.87501	72.05441	35.88131
378	152.019	157.6683	58.26039	78.65212	70.84754	34.93081
378.5	149.6614	154.903	57.46758	77.35435	69.84475	34.21012
379	147.5222	152.328	56.85405	76.23184	69.01453	33.6505
379.5	145.3066	149.6119	56.37013	75.17913	68.50044	33.01168
380	142.7604	146.7312	55.89226	73.72379	67.86337	32.43683
380.5	139.8874	143.8648	55.04362	71.8374	66.73611	31.65994
381	136.6738	140.93	54.12635	69.67186	65.32142	31.07493
381.5	133.4347	138.3078	52.8799	67.3207	63.71173	30.54157
382	129.9832	135.7073	51.63807	65.12691	61.99852	29.98114
382.5	126.5571	133.3278	50.67696	63.20086	60.56077	29.31209
383	123.2674	131.0068	49.85118	61.53721	59.26861	28.58117
383.5	120.5643	128.9526	49.1776	60.34856	58.49507	28.15445
384	118.1479	127.2985	48.62417	59.72671	58.05259	27.91832
384.5	116.2289	125.8255	48.33316	59.172	57.96957	27.93316
385	114.7573	124.2071	48.03653	58.61468	57.82596	27.68987
385.5	113.148	122.3091	47.67469	58.02169	57.28715	27.53002
386	111.4948	120.2919	46.81608	57.13628	56.28829	27.25327
386.5	109.6153	118.033	45.73273	56.16892	54.89832	26.93194
387	107.4526	115.7769	44.44512	54.98673	53.28607	26.41402
387.5	104.9314	113.1311	43.0603	53.753	51.48271	25.45457
388	102.2359	110.4951	41.56399	52.53763	49.88699	24.34769
388.5	99.64035	107.9302	40.17668	51.44722	48.40083	23.42255
389	97.3319	105.9323	39.1009	50.34602	47.2915	22.68511
389.5	95.6571	104.1743	38.45783	49.34457	46.61731	22.18712

390	94.36942	102.6154	38.43624	48.76159	45.93099	22.07637
390.5	93.2	101.1741	38.43794	48.11671	45.13169	22.13034
391	92.00152	99.8752	38.52084	47.46827	44.4493	22.33327
391.5	90.80435	98.8336	38.4691	46.7391	43.9774	22.68666
392	89.2355	97.49501	38.21462	45.92843	43.51032	22.62765
392.5	87.19711	96.23303	37.61164	45.27737	43.19246	22.37774
393	85.01509	94.70101	36.80137	44.58829	42.77732	22.21102
393.5	82.73695	93.17515	35.64115	43.67775	42.26	21.66452
394	80.66917	91.58293	34.45384	42.64447	42.0875	21.01634
394.5	79.39704	90.42151	33.83494	42.23548	42.01501	20.76988
395	78.54937	89.42081	33.4279	41.95068	41.69231	20.7966
395.5	77.6624	88.55173	33.10539	41.31476	41.11423	20.66207
396	77.0839	87.77375	32.86456	40.45105	40.51237	20.59776
396.5	77.48424	86.82099	31.95824	39.58667	39.45749	20.31859
397	78.65377	85.91611	30.19085	39.045	38.10615	20.02522
397.5	78.98101	84.5326	28.94177	38.33993	37.03506	19.8815
398	76.56215	82.77753	28.88878	36.84406	36.55609	19.10898
398.5	70.49667	80.32785	30.98216	34.4771	37.80637	17.87836
399	61.11612	78.14919	36.36196	31.88104	41.34283	16.79463
399.5	48.03913	75.83293	45.04889	29.01777	47.23055	15.82584
400	30.90044	73.40729	56.46963	25.36405	54.76035	14.4127

Table 1. Data from Fig. 1 A

Temperature / Wavelength (nm)	25 °C	40 °C	60 °C	70 °C	80 °C
390	999.999	999.999	999.999	969.9992	999.999
390.5	999.999	999.999	999.999	969.9992	999.999
391	999.999	999.999	999.999	969.9992	999.999
391.5	999.999	999.999	999.999	969.8359	999.999
392	999.999	999.999	999.999	969.2889	999.999
392.5	999.999	999.999	999.999	968.5176	999.999
393	999.999	999.999	999.999	968.072	999.999
393.5	999.999	999.999	999.999	968.7734	999.999
394	999.999	999.999	999.999	971.509	999.999
394.5	999.999	999.999	999.999	976.7334	999.999
395	1002.743	1003.247	1003.272	984.2828	999.999
395.5	1010.914	1011.602	1011.642	993.2257	999.999
396	1021.096	1021.674	1021.776	1001.473	1002.713
396.5	1027.234	1027.312	1027.414	1005.756	1010.788
397	1022.285	1021.349	1021.443	1002.403	1021.063
397.5	999.5831	997.156	997.2032	987.9298	1027.818
398	952.5992	948.3626	948.2331	959.616	1023.597
398.5	878.7171	872.5057	871.9691	916.04	999.9379
399	777.3273	769.2163	768.0684	857.0396	952.2033
399.5	655.7454	647.0093	645.114	783.8311	877.3581
400	528.1974	519.4263	516.7397	698.986	774.7371
400.5	405.9779	397.9029	394.5927	605.9316	651.7387
401	298.0544	291.3192	287.5045	508.6455	522.7417
401.5	210.6096	205.4738	201.5012	411.6865	399.7855
402	146.7958	143.2262	139.5389	319.6148	293.0216
402.5	104.3176	102.0455	99.0148	236.578	208.86
403	79.52112	78.04104	75.84226	166.0211	147.7285
403.5	64.83419	63.40719	62.09532	110.041	109.261
404	54.48116	53.24387	52.77134	69.32702	89.18905
404.5	47.69738	46.39033	46.60074	43.02586	79.31989
405	43.40994	41.99353	42.75079	28.74355	73.27419
405.5	40.87461	39.46787	40.47684	23.21952	69.63317
406	39.75143	38.37917	39.45619	23.26024	67.26383
406.5	40.18775	38.85007	39.94976	26.08771	66.18417
407	40.68145	39.34452	40.48005	29.66643	66.80538
407.5	41.13419	39.78016	40.93252	32.82849	67.32389
408	41.56639	40.12817	41.32568	35.02717	67.69621
408.5	41.91705	40.4121	41.67295	36.37431	67.99273
409	42.26637	40.66292	41.98758	37.32602	68.30914
409.5	42.6333	40.94967	42.3135	38.18542	68.67357
410	43.01157	41.29696	42.66488	38.98663	69.12191
410.5	43.46407	41.71388	43.06385	39.74807	69.70773

411	43.98951	42.19304	43.5524	40.46162	70.35083
411.5	44.6031	42.75134	44.16152	41.15749	71.12108
412	45.25685	43.42465	44.88756	41.96078	71.9752
412.5	46.0019	44.14193	45.7265	42.87871	72.90768
413	46.78486	44.88661	46.66742	43.90859	73.92736
413.5	47.67132	45.65773	47.68354	45.04841	75.04921
414	48.60569	46.49543	48.70524	46.29493	76.26399
414.5	49.59356	47.38803	49.72447	47.64513	77.58491
415	50.67504	48.3739	50.76523	49.09766	79.06699
415.5	51.88075	49.44457	51.80815	50.65731	80.64214
416	53.2028	50.60642	52.92034	52.32273	82.3669
416.5	54.58523	51.86817	54.07922	54.10569	84.16214
417	56.00381	53.19172	55.32067	56.00985	86.07402
417.5	57.4615	54.54568	56.61219	58.64372	88.10177
418	59.04324	55.92756	58.02079	61.0378	90.21004
418.5	60.71357	57.37672	59.53313	63.23793	92.45228
419	62.47348	58.89217	61.13548	65.28272	94.83817
419.5	64.29709	60.49085	62.8776	67.21372	97.38666
420	66.23082	62.16886	64.71081	69.0773	100.1039
420.5	68.27349	63.97819	66.71523	70.91245	103.0132
421	70.42527	65.88675	68.85177	72.76585	106.0225
421.5	72.61361	67.8485	71.06275	74.66886	109.1873
422	74.8395	69.85645	73.30166	76.67087	112.4401
422.5	77.14163	71.92957	75.58387	78.82236	115.7252
423	79.56615	74.10355	77.90175	81.16351	119.0911
423.5	82.12557	76.44029	80.27201	83.73514	122.6046
424	84.7885	78.89771	82.72477	86.58408	126.2386
424.5	87.55812	81.48104	85.22831	89.76302	129.9938
425	90.36841	84.22496	87.84445	93.32547	133.9085
425.5	93.24725	87.1347	90.63819	97.32372	137.9542
426	96.22515	90.15698	93.52902	101.7885	142.2778
426.5	99.301	93.23708	96.49763	106.7673	146.7143
427	102.4685	96.33639	99.58377	111.2696	151.2192
427.5	105.7929	99.42922	102.7446	115.8715	155.8507
428	109.2415	102.5868	105.963	120.5518	160.6474
428.5	112.8619	105.7856	109.2686	125.6047	165.5479
429	116.6166	108.957	112.591	130.5382	170.4743
429.5	120.4036	112.1497	115.909	135.3848	175.3802
430	124.176	115.4373	119.2835	140.1837	180.2683
430.5	128.0103	118.819	122.6661	144.9764	185.3165
431	131.841	122.305	126.1047	150.1364	190.3744
431.5	135.7321	125.9322	129.709	155.2345	195.5705
432	139.7741	129.5992	133.3995	160.3258	201.0337
432.5	143.8655	133.3517	137.1687	165.4477	206.8274
433	148.0415	137.1853	141.0491	170.6348	212.9256
433.5	152.3015	141.0319	145.0588	175.9016	219.3545

434	156.6253	144.9302	149.121	181.2528	226.0026
434.5	160.9447	148.8268	153.1975	186.6845	232.8426
435	165.3631	152.7755	157.2901	192.1861	239.8201
435.5	169.7292	156.8295	161.4126	197.7707	246.6394
436	174.0453	161.0671	165.7434	203.4726	253.1407
436.5	178.4219	165.3518	170.2217	209.3355	259.387
437	182.7867	169.6574	174.7965	215.7085	265.5218
437.5	187.1412	174.0007	179.425	222.3285	271.5526
438	191.4958	178.2379	184.1225	228.3386	277.6636
438.5	195.9583	182.5	188.8263	234.7019	283.9239
439	200.4875	186.7558	193.4882	241.2968	290.421
439.5	205.2575	190.9068	197.974	247.7836	297.3048
440	210.2183	195.102	202.3994	254.2667	304.598
440.5	215.2912	199.5249	206.8897	260.2798	311.9206
441	220.5301	203.9938	211.3859	266.0014	319.1989
441.5	225.8406	208.5114	215.9528	271.4986	326.4854
442	231.1293	213.1579	220.5449	276.8211	333.7617
442.5	236.4171	217.8445	225.1418	282.0292	340.956
443	241.7063	222.6095	229.7977	287.1784	348.1028
443.5	246.9822	227.4905	234.5925	292.3456	355.0398
444	252.1551	232.2865	239.3313	297.6192	362.0816
444.5	257.2456	237.1408	244.1395	303.0891	369.5123
445	262.3391	242.2178	249	308.8796	376.8988
445.5	267.4254	247.2821	253.8734	315.0858	384.192
446	272.4586	252.1769	258.8513	321.79	391.4094
446.5	277.3673	256.9603	263.8457	328.5461	398.7799
447	282.2431	261.61	268.8084	335.4185	406.0726
447.5	287.1233	266.1165	273.6688	342.8675	413.4721
448	292.1497	270.505	278.6193	350.2417	420.7629
448.5	297.0849	274.7244	283.4658	357.3591	427.8639
449	301.7772	278.9217	288.2185	364.4229	434.991
449.5	306.4724	283.2422	292.9316	371.3575	441.8782
450	311.256	287.6544	297.4827	378.1046	448.5176
450.5	316.18	292.0487	302.0318	384.797	454.8926
451	321.2221	296.4402	306.4274	391.4444	461.2366
451.5	326.263	300.8282	310.7445	398.073	467.5168
452	331.2848	305.2387	315.0022	404.668	473.9911
452.5	336.484	309.4544	319.3529	411.2346	480.6583
453	341.608	313.5647	323.797	417.7671	487.3369
453.5	346.2729	317.5708	328.2003	424.2313	494.1361
454	350.6937	321.5952	332.6687	430.6289	500.88
454.5	354.932	325.7079	337.1204	436.9587	507.4271
455	359.0017	329.8458	341.5631	443.2022	513.7467
455.5	363.214	334.1052	345.7762	449.3229	519.7557
456	367.3413	338.2107	349.8981	455.2896	525.3938
456.5	371.5865	342.237	353.8726	461.0505	530.9202

457	376.0198	346.1243	357.6336	466.6086	536.3199
457.5	380.4984	349.8655	361.252	471.964	541.5322
458	384.8028	353.3436	364.6137	477.0768	546.8507
458.5	388.973	356.7105	367.8577	481.9489	552.1055
459	392.9424	359.8989	371.0361	486.6032	557.0133
459.5	396.5668	362.9321	374.1348	491.0634	561.5828
460	400.1744	365.9957	377.1472	495.3489	565.8236
460.5	403.3573	368.8556	380.1805	499.4778	569.7865
461	406.2836	371.4396	383.1715	503.4582	573.7296
461.5	409.119	374.0059	386.0692	507.3423	577.2743
462	411.6512	376.6219	388.9657	511.1658	580.5536
462.5	414.1274	379.0777	391.7274	514.9268	584.1185
463	416.5767	381.6015	394.3558	518.639	587.895
463.5	419.0267	384.2084	396.9699	522.3078	591.6884
464	421.4153	386.8651	399.3407	525.9123	595.4401
464.5	423.9367	389.6349	401.5395	529.4067	599.2049
465	426.2276	392.2569	403.6331	532.7241	602.9024
465.5	428.3183	394.5117	405.4803	535.8268	606.4981
466	430.4229	396.5995	407.0775	538.6974	609.5247
466.5	432.1508	398.6093	408.6321	541.2648	611.9391
467	433.8106	400.2811	410.1087	543.4811	613.9625
467.5	435.4074	401.5999	411.5834	545.3439	615.6663
468	436.83	402.7873	413.1293	546.8731	617.0227
468.5	438.1107	403.8389	414.5728	548.0992	617.9778
469	439.4864	404.756	415.9956	549.0507	618.7329
469.5	440.781	405.4611	417.3014	549.7589	619.5608
470	441.8057	405.8881	418.4436	550.2931	620.1281
470.5	442.8922	406.1938	419.3178	550.7023	620.4191
471	443.6899	406.6488	419.9774	550.9768	620.7382
471.5	444.303	407.1671	420.4082	551.1034	620.9045
472	444.8361	407.4835	420.634	551.083	621.0395
472.5	445.1027	407.8699	420.7015	550.9109	621.0693
473	445.0227	408.2401	420.5484	550.5859	620.8426
473.5	444.8111	408.6165	420.3357	550.0978	620.4161
474	444.5269	408.845	419.8171	549.4564	619.9798
474.5	443.9996	408.8391	419.316	548.726	619.0735
475	443.4863	408.4969	418.8727	547.9277	617.9406
475.5	443.0437	407.8827	418.4171	547.0727	616.883
476	442.6992	407.0087	418.0588	546.1824	615.8387
476.5	442.585	405.8022	417.8187	545.2846	614.7849
477	442.5153	404.5855	417.6492	544.4072	613.8831
477.5	442.3859	403.5347	417.3699	543.5751	613.1027
478	442.2883	402.7991	417.0669	542.7674	612.4999
478.5	442.3401	402.4814	416.3248	541.9774	612.0458
479	442.1603	402.5012	415.4	541.2241	611.3465
479.5	441.5701	402.7034	414.4714	540.4484	610.548

480	440.698	402.8884	413.4956	539.6122	609.6369
480.5	439.6877	402.6081	412.5568	538.6786	608.7773
481	438.4601	401.8248	411.5496	537.6104	607.6972
481.5	436.9932	400.5424	410.6479	536.3455	606.4334
482	435.3504	398.853	409.8	534.8753	604.8662
482.5	433.7682	396.9623	409.0966	533.1781	603.3198
483	432.4872	395.2265	408.0686	531.2387	601.6052
483.5	431.3605	393.7507	406.7944	529.0714	599.4385
484	430.1613	392.6339	405.3734	526.6715	597.1509
484.5	428.9175	391.8916	403.9143	524.0683	594.4908
485	427.915	391.061	402.3677	521.3326	591.6494
485.5	426.877	390.2009	400.5945	518.5463	588.5805
486	425.5535	389.2756	398.8159	515.7404	585.6556
486.5	423.9304	388.1286	397.2371	512.9591	582.4984
487	422.0966	386.6539	395.8924	510.2213	579.3809
487.5	420.1869	385.045	394.4685	507.5473	576.5708
488	418.1953	383.2521	393.0016	504.9332	574.036
488.5	416.0355	381.4478	391.3726	502.3426	571.8669
489	413.6539	379.6777	389.818	499.7336	569.9487
489.5	411.383	377.6011	388.1253	497.0787	567.7418
490	409.4095	375.3992	386.1462	494.3749	565.166

Table 2. Data from Fig. 1 B

Sample	Area	Percent (%)
**25 °C**	17391.3	17.489
**30 °C**	24745.3	14.817
**40 °C**	24335.15	21.082
**50 °C**	23355	20.732
**60 °C**	4107.598	19.897
**70 °C**	2914.678	3.499
**80 °C**	20528.76	2.483

Table 3. Data from Fig. 2 A

Sample	Area	Percent (%)
**25 °C,DTT+, GA-**	0	0
**25 °C,DTT+, GA+**	0	0
**60 °C,DTT+, GA-**	0	0
**60 °C,DTT+, GA+**	0	0
**25 °C,DTT-, GA-**	33350.53	42.762
**25 °C,DTT-, GA+**	10524.9	13.495
**60 °C,DTT-, GA-**	19573.82	25.098
**60 °C,DTT-, GA+**	14541.29	18.645

Table 4. Data from Fig. 2 D

Size Distribution Report by Intensity

v2.2

Sample Details

**Sample Name:**	25@96.2.12 1
**SOP Name:**	size.sop
**General Notes:**	

**File Name:**	kh.afshar	**Dispersant Name:**	PBS
**Record Number:**	1	**Dispersant RI:**	1.334
**Material RI:**	1.33	**Viscosity (cP):**	0.9200
**Material Absorbtion:**	0.000	**Measurement Date and Time:**	Wednesday, May 3, 2017 3:4...

System

**Temperature (°C):**	25.0	**Duration Used (s):**	60
**Count Rate (kcps):**	184.6	**Measurement Position (mm):**	5.50
**Cell Description:**	Clear disposable zeta cell	**Attenuator:**	9

Results

			**Size (d.nm):**	**% Intensity:**	**St Dev (d.n...**
**Z-Average (d.nm):**	102.7	**Peak 1:**	186.5	60.5	59.53
**PdI:**	0.270	**Peak 2:**	22.48	39.5	6.368
**Intercept:**	0.771	**Peak 3:**	0.000	0.0	0.000

Result quality : **Refer to quality report**

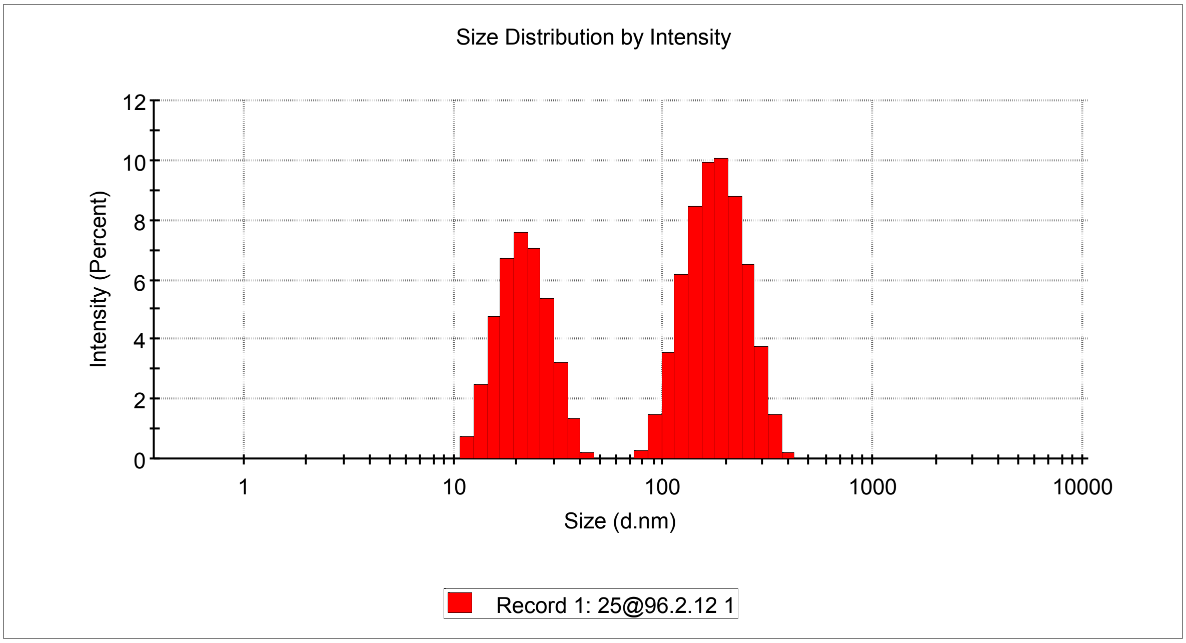

Size Distribution Report by Intensity

v2.2

Sample Details

Sample Name: 50@96.2.12 1

SOP Name: size.sop

General Notes:

**File Name:**	kh.afshar	**Dispersant Name:**	PBS
**Record Number:**	2	**Dispersant RI:**	1.334
**Material RI:**	1.33	**Viscosity (cP):**	0.9200
**Material Absorbtion:**	0.000	**Measurement Date and Time:**	Wednesday, May 3, 2017 3:5...

System

**Temperature (°C):**	25.0	**Duration Used (s):**	60
**Count Rate (kcps):**	296.4	**Measurement Position (mm):**	5.50
**Cell Description:**	Clear disposable zeta cell	**Attenuator:**	8

Results

			Size (d.nm):	% Intensity:	St Dev (d.n...
**Z-Average (d.nm):**	101.2	**Peak 1:**	336.5	76.5	213.7
**PdI:**	1.000	**Peak 2:**	29.86	19.9	13.01
**Intercept:**	0.669	**Peak 3:**	4532	3.5	868.1

Result quality : **Refer to quality report**

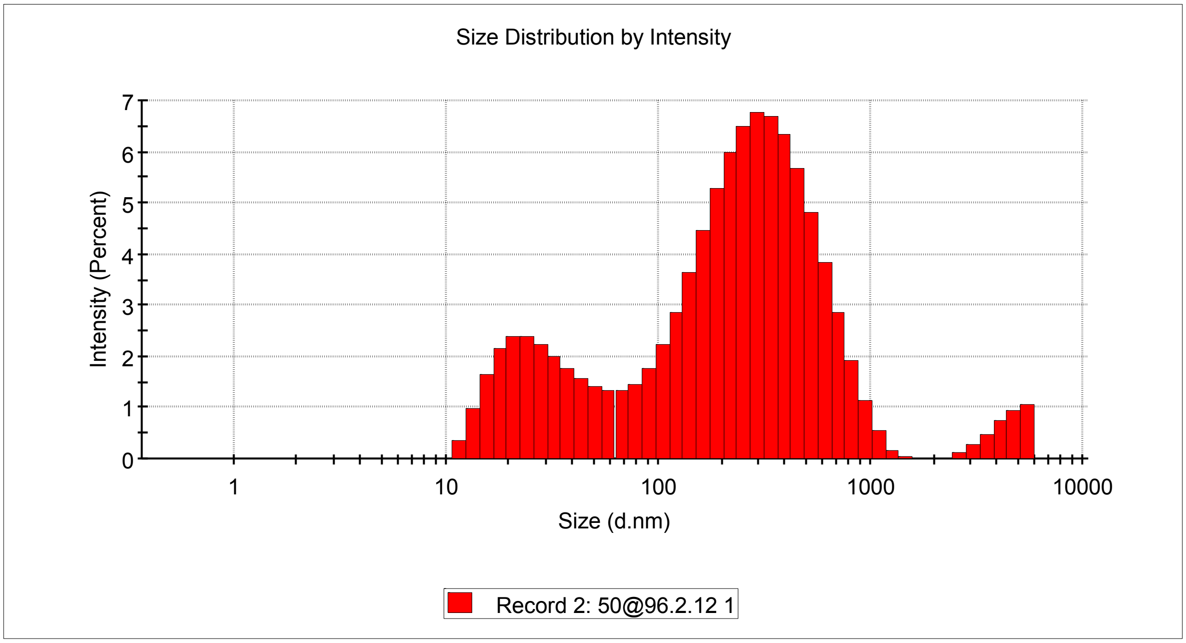

Size Distribution Report by Intensity
v2.2

Sample Details

**Sample Name:**	70@96.2.12 1
**SOP Name:**	size.sop
**General Notes:**	

**File Name:**	kh.afshar	**Dispersant Name:**	Water
**Record Number:**	3	**Dispersant RI:**	1.330
**Material RI:**	1.33	**Viscosity (cP):**	0.8872
**Material Absorbtion:**	0.000	**Measurement Date and Time:**	Wednesday, May 3, 2017 4:1...

System

**Temperature (°C):**	25.0	**Duration Used (s):**	70
**Count Rate (kcps):**	148.3	**Measurement Position (mm):**	5.50
**Cell Description:**	Clear disposable zeta cell	**Attenuator:**	6

Results

			**Size (d.nm):**	**% Intensity:**	**St Dev (d.n...**
**Z-Average (d.nm):**	1.178e4	**Peak 1:**	711.1	100.0	11.19
**PdI:**	1.000	**Peak 2:**	0.000	0.0	0.000
**Intercept:**	0.803	**Peak 3:**	0.000	0.0	0.000

Result quality : **Refer to quality report**

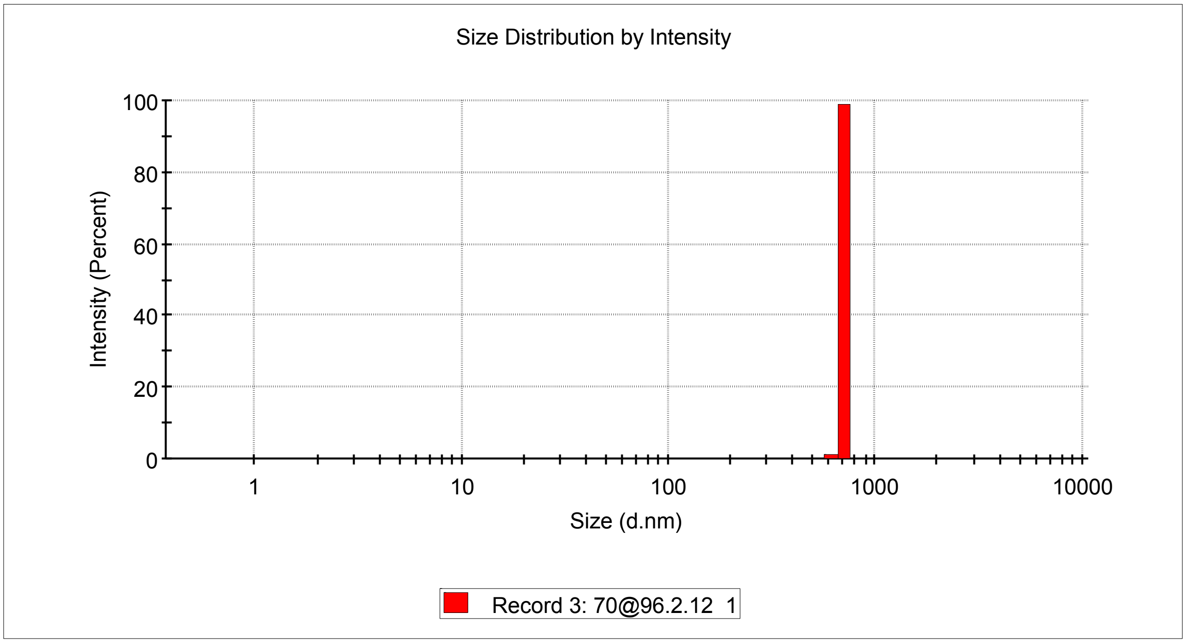

Size Distribution Report by Intensity

v2.2

Sample Details

Sample Name: 80@96.2.12 1 1

SOP Name: size.sop

General Notes:

**File Name:**	kh.afshar	**Dispersant Name:**	Water
**Record Number:**	4	**Dispersant RI:**	1.330
**Material RI:**	1.33	**Viscosity (cP):**	0.8872
**Material Absorbtion:**	0.000	**Measurement Date and Time:**	Wednesday, May 3, 2017 4:3...

System

**Temperature (°C):**	25.0	**Duration Used (s):**	80
**Count Rate (kcps):**	134.0	**Measurement Position (mm):**	5.50
**Cell Description:**	Clear disposable zeta cell	**Attenuator:**	6

Results

			**Size (d.nm):**	**% Intensity:**	**St Dev (d.n...**
**Z-Average (d.nm):**	7910	**Peak 1:**	2305	100.0	0.000
**PdI:**	0.370	**Peak 2:**	0.000	0.0	0.000
**Intercept:**	0.797	**Peak 3:**	0.000	0.0	0.000

Result quality : **Refer to quality report**

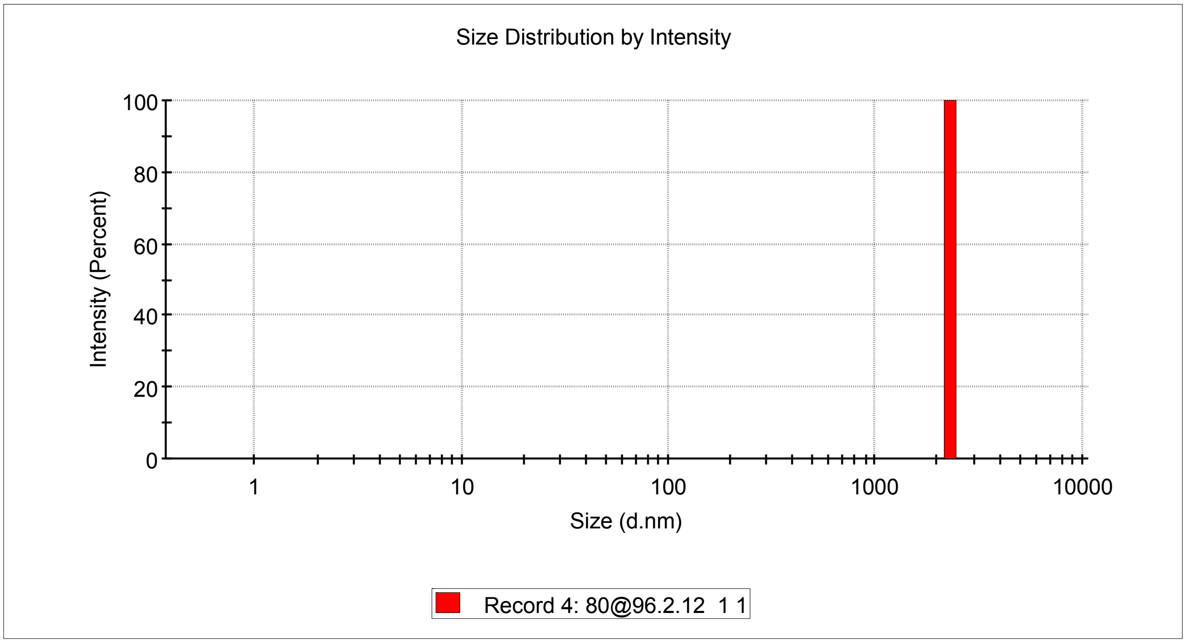

H_2O_2 (mM) / Wavelength (nm)	0	2.5	5	15	20	30	40	80	160
300	139.963	114.925	118.571	107.429	108.683	86.849	83.55	10.196	-18.093
300.5	138.449	116.984	119.238	107.321	106.336	86.299	81.424	11.588	-16.293
301	143.337	123.586	123.862	113.038	111.933	93.596	86.014	14.555	-14.38
301.5	148.803	128.917	129.603	115.749	117.378	95.003	89.859	15.177	-12.87
302	155.065	129.113	132.475	118.658	120.077	98.679	93.44	18.88	-12.183
302.5	158.946	137.171	139.503	125.692	124.767	103.282	97.184	20.926	-10.154
303	162.065	138.659	141.913	125.538	127.648	106.559	99.592	20.412	-10.302
303.5	168.135	145.96	149.15	135.122	133.585	113.764	106.714	25.899	-7.576
304	171.542	146.144	149.327	134.364	136.303	112.358	106.094	24.078	-7.721
304.5	185.774	155.372	159.724	147.105	146.522	121.513	115.418	29.863	-4.397
305	178.838	154.07	158.905	145.34	145.248	122.32	115.161	29.7	-4.116
305.5	189.517	165.589	168.186	152.841	153.906	129.544	121.87	33.309	-1.6
306	194.662	166.137	170.79	154.264	152.915	130.407	124.071	34.454	-0.761
306.5	201.595	170.194	179.705	160.98	158.009	135.092	129.253	37.901	1.243
307	207.064	179.301	181.553	164.515	164.402	139.241	131.886	41.352	3.115
307.5	212.105	182.803	186.633	169.879	169.396	144.508	138.884	43.382	4.28
308	215.99	188.554	189.799	174.905	171.948	148.512	141.233	44.974	6.097
308.5	221.703	190.332	199.308	181.726	176.36	152.83	145.478	47.317	7.892
309	227.992	196.688	198.149	182.782	185.384	154.196	147.74	48.638	8.332
309.5	228.518	200.331	205.008	188.592	185.339	158.551	151.944	51.283	10.46
310	236.931	203.153	208.204	190.898	193.659	163.796	155.509	54.928	11.481
310.5	237.816	208.927	214.074	194.115	195.739	166.476	157.853	55.958	13.021
311	243.224	212.793	220.162	201.508	201.798	171.471	161.39	58.608	14.439
311.5	249.032	216.267	223.716	203.475	205.008	175.465	166.208	60.746	16.168
312	254.432	223.962	225.993	209.201	209.902	178.596	170.046	61.962	17.334
312.5	258.685	226.1	227.414	212.916	211.013	182.336	174.521	64.599	19.19
313	262.114	228.15	232.792	219.952	216.557	186.885	177.643	69.529	20.666
313.5	267.535	235.217	238.547	224.124	223.338	192.543	185.734	70.167	22.9
314	271.717	239.271	241.271	226.08	227.402	194.343	187.688	72.255	24.598
314.5	275.581	244.107	248.022	229.633	229.681	199.687	190.97	73.554	27.436

315	281.705	245.688	252.857	231.273	233.769	200.71	192.608	76.217	27.977
315.5	283.621	249.644	255.974	237.558	236.514	203.142	196.672	78.188	28.245
316	286.272	252.622	258.362	236.688	238.056	207.396	197.526	79.537	29.156
316.5	296.002	261.258	264.364	245.619	247.349	211.191	203.3	83.384	32.468
317	295.563	260.346	264.504	246.947	245.289	212.693	203.371	81.01	30.675
317.5	299.742	265.708	269.789	251.263	251.97	218.108	208.317	85.116	32.324
318	297.589	265.535	271.321	251.908	248.881	216.289	209.07	86.362	33.86
318.5	303.856	271.78	275.202	254.821	257.076	223.652	216.099	87.343	35.794
319	305.507	270.615	275.788	258.686	260.066	223.441	214.738	89.03	36.313
319.5	311.319	276.324	282.468	262.572	260.331	223.271	219.412	91.434	38.074
320	313.932	278.447	284.27	266.984	264.228	228.894	220.206	92.768	38.352
320.5	317.659	280.286	289.313	269.455	268.179	234.524	225.62	95.127	40.039
321	318.872	283.779	289.843	268.454	268.389	236.947	224.662	95.983	41.43
321.5	320.58	285.661	291.25	271.621	271.595	236.7	227.284	95.835	42.549
322	323.04	285.837	297.203	275.218	275.197	237.766	231.345	99.265	43.013
322.5	327.911	290.871	299.518	279.51	277.165	240.852	234.596	100.715	43.697
323	331.153	294.764	300.154	281.159	280.143	244.847	236.793	105.361	45.539
323.5	333.038	299.401	301.409	281.031	283.627	243.168	237.868	103.965	46.673
324	337.441	297.381	301.765	282.739	283.324	247.97	237.328	104.98	46.935
324.5	334.555	300.315	306.092	285.972	282.861	248.116	241.132	106.881	48.37
325	338.52	300.349	305.834	289.13	288.442	249.19	243.714	107.598	48.629
325.5	337.852	301.659	308.62	291.243	289.674	250.218	243.226	106.972	49.068
326	340.005	307.484	313.878	289.646	291.064	251.927	244.876	109.415	51.815
326.5	341.085	305.534	312.918	290.849	290.75	251.594	245.088	109.592	50.551
327	347.64	306.046	313.427	294.584	292.962	256.146	248.391	110.217	50.832
327.5	344.734	308.257	312.749	292.555	295.512	255.938	249.101	113.259	51.562
328	348.208	306.719	316.981	293.7	295.789	256.427	249.297	112.96	53.101
328.5	345.75	307.419	319.203	298.351	297.958	259.166	250.495	114.583	55.061
329	344.943	312.195	319.586	297.627	297.982	258.465	251.829	114.415	53.808
329.5	346.828	312.643	316.601	298.789	300.768	259.184	255.704	115.021	54.676
330	349.406	310.385	319.461	300.106	296.256	260.403	251.914	116.253	57.371
330.5	352.489	311.699	320.141	297.42	299.778	261.29	253.698	116.719	57.133
331	345.979	312.582	318.637	299.997	302.062	260.549	253.25	118.652	57.565

331.5	344.925	314.7	320.88	297.939	301.919	259.999	252.889	115.967	56.219
332	346.701	309.944	320.853	301.311	300.955	264.086	255.312	119.738	57.413
332.5	349.128	313.828	319.416	300.625	301.621	264.839	256.549	119.371	58.884
333	348.03	313.036	317.518	300.034	299.877	262.482	252.951	118.426	58.389
333.5	345.89	312.141	318.931	300.811	297.899	260.114	252.508	118.898	58.093
334	347.739	312.998	318.001	300.325	302.62	261.047	254.343	119.872	59.768
334.5	345.38	309.31	315.975	300.602	300.306	260.089	255.903	121.053	59.631
335	347.853	312.222	318.13	300.492	297.111	261.935	254.012	118.594	59.163
335.5	346.017	311.95	316.984	297.226	299.478	262.486	252.483	120.358	60.377
336	345.645	312.471	317.811	296.995	293.887	260.529	253.8	119.799	60.162
336.5	341.851	308.762	313.218	293.005	298.201	259.438	250.14	119.252	58.46
337	341.63	308.282	312.406	297.43	291.11	258.399	250.651	119.23	59.249
337.5	339.852	304.224	312.978	290.908	293.441	256.703	251.493	118.114	60.426
338	337.671	304.218	310.18	291.412	289.484	253.207	247.933	118.221	58.648
338.5	337.747	303.338	309.496	290.422	290.253	252.514	247.965	117.807	59.318
339	332.671	301.841	311.035	290.065	289.783	250.153	247.286	117.802	59.833
339.5	333.544	299.176	307.649	289.098	286.468	249.856	246.848	117.057	59.422
340	331.453	298.751	304.2	289.477	288.747	250.662	245.016	117.217	60.298
340.5	327.303	294.362	304.541	283.064	284.535	247.619	244.559	115.792	58.801
341	331.076	295.559	302.512	280.493	280.398	248.375	242.867	114.735	58.39
341.5	319.919	290.846	299.908	279.559	281.602	247.219	241.653	115.569	58.173
342	326.535	293.883	299.738	282.343	280.213	244.56	239.291	115.513	59.983
342.5	318.239	287.305	295.526	277.846	277.526	242.03	235.308	113.944	58.614
343	317.794	291.005	296.89	275.393	276.079	241.249	237.552	115.168	59.048
343.5	315.896	286.6	291.744	270.745	276.024	238.431	235.178	113.954	57.476
344	314.152	283.147	289.05	271.292	271.729	238.629	232.518	112.739	57.448
344.5	308.969	280.771	287.121	269.004	270.8	234.346	231.733	111.973	56.487
345	308.049	278.654	286.884	267.923	269.132	233.364	228.186	111.061	55.663
345.5	302.901	274.989	279.346	264.817	264.498	229.984	226.254	110.055	56.025
346	298.091	271.443	280.941	260.081	261.561	230.665	222.573	109.715	55.839
346.5	297.947	270.48	279.428	259.845	261.76	228.633	222.678	108.433	56.473
347	294.675	269.961	272.913	257.457	256.956	225.727	221.674	109.694	55.548
347.5	291.159	267.861	275.414	259.182	254.725	224.265	218.707	107.059	54.602

348	288.401	262.932	271.259	254.001	251.968	220.514	216.911	107.08	53.963
348.5	284.619	260.29	269.432	251.374	250.524	217.511	213.721	105.466	54.2
349	285.757	258.371	263.3	246.751	248.546	215.935	214.023	104.539	52.038
349.5	280.226	255.904	262.466	245	244.908	213.552	211.656	104.189	52.575
350	280.937	252.243	259.126	244.758	245.006	212.218	205.93	102.161	52.264
350.5	275.242	252.016	256.645	238.96	240.595	210.451	206.183	100.167	51.287
351	272.813	249.998	253.803	239.463	241.504	210.077	205.991	98.168	52.042
351.5	266.789	245.295	248.502	235.161	235.702	204.949	201.375	98.862	50.233
352	268.811	245.117	247.305	233.835	232.51	206.345	198.994	97.297	51.501
352.5	262.512	239.702	246.262	228.782	229.401	199.661	193.556	95.291	50.362
353	260.878	238.709	243.792	227.451	227.504	196.503	194.353	94.993	49.725
353.5	257.633	233.852	238.68	223.843	223.133	195.113	192.13	95.686	48.555
354	257.054	231.149	237.552	222.281	223.491	191.343	190.978	93.04	47.076
354.5	251.658	228.507	234.723	219.684	220.65	190.892	187.662	92.225	47.687
355	249.533	224.783	233.028	215.605	218.204	188.442	184.838	92.269	46.994
355.5	244.158	223.189	227.581	212.012	213.851	185.539	183.296	89.784	46.315
356	244.374	221.657	228.556	211.462	212.09	182.602	181.133	91.68	46.011
356.5	237.728	218.122	221.758	208.586	208.574	179.13	180.616	89.366	45.332
357	233.616	212.961	217.224	205.028	204.143	177.682	175.906	86.646	43.35
357.5	230.157	210.275	216.546	203.446	200.802	176.375	172.921	86.59	43.802
358	228.521	205.316	209.814	199.799	199.161	174.041	173.014	82.727	43.062
358.5	224.412	200.728	211.056	196.989	195.087	171.827	170.724	84.103	41.877
359	223.982	205.094	208.757	195.486	193.732	170.596	166.99	83.083	41.823
359.5	217.761	200.025	202.925	190.354	191.403	166.502	165.142	81.561	41.607
360	215.123	193.702	201.354	185.837	188.499	162.691	161.485	79.744	39.532
360.5	215.168	193.466	198.751	184.528	186.451	163.765	160.105	81.253	40.003
361	210.605	189.21	195.079	182.796	181.812	156.949	157.66	79.759	39.072
361.5	208.092	185.976	193.219	179.778	180.428	157.213	156.437	79.417	39.819
362	200.486	183.25	189.072	178.752	176.312	153.985	153.214	78.125	38.521
362.5	197.313	183.644	186.263	175.968	175.104	153.51	150.816	76.344	38.914
363	194.965	178.446	182.566	172.216	173.735	150.296	148.349	74.446	37.348
363.5	193.502	176.222	181.076	168.026	170.363	148.426	146.424	73.305	36.822
364	190.14	173.91	179.192	166.292	166.811	145.002	143.972	71.375	35.47

364.5	187.665	171.603	175.506	166.1	165.856	144.667	142.029	71.276	36.096
365	185.851	170.253	172.247	164.512	164.2	143.096	139.046	71.523	35.729
365.5	179.768	166.563	169.118	158.579	158.143	136.766	137.682	68.756	33.997
366	177.407	164.067	168.954	155.827	157.105	137.587	135.778	67.469	33.693
366.5	174.714	162.241	167.606	154.013	153.771	135.147	132.522	66.67	33.892
367	174.358	158.18	163.709	151.902	154.297	134.132	131.852	66.003	32.095
367.5	168.902	154.497	158.637	148.325	150.124	131.008	128.893	65.381	32.706
368	167.443	154.847	158.113	148.113	147.846	128.698	127.002	64.445	31.719
368.5	163.951	148.99	154.034	144.76	145.619	125.767	125.068	62.647	31.342
369	160.754	148.279	152.972	143.024	144.519	125.929	123.421	62.39	30.355
369.5	155.765	144.576	149.646	139.435	141.071	122.107	118.752	61.53	29.761
370	157.511	144.01	146.365	139.555	139.534	121.795	119.594	59.965	29.02
370.5	152.148	140.679	143.083	134.618	134.935	117.975	115.755	59.742	29.188
371	152.292	138.585	143.257	136.642	134.825	117.361	115.863	58.59	30.019
371.5	147.444	133.958	138.203	131.055	128.298	112.65	111.438	55.413	26.921
372	147.033	136.408	139.224	129.227	129.256	112.956	111.543	56.388	28.13
372.5	141.411	131.296	133.697	127.382	125.276	109.928	108.649	55.072	27.325
373	141.054	132.712	133.493	125.856	127.153	111.216	108.77	56.017	27.778
373.5	136.742	127.882	130.075	121.25	123.183	106.947	103.819	55.133	26.638
374	135.32	124.712	129.431	121.056	120.945	105.404	104.928	52.049	24.408
374.5	132.859	120.695	125.738	118.273	117.632	106.113	101.758	52.142	24.773
375	132.155	122.032	125.16	117.175	119.089	103.545	102.371	52.638	25.257
375.5	127.269	117.883	122.834	113.635	114.757	100.235	99.607	49.635	23.852
376	126.235	117.972	118.556	112.652	111.808	98.231	97.156	48.815	24.011
376.5	124.389	114.532	119.095	109.167	110.817	95.502	95.432	48.392	22.572
377	120.936	113.923	117.384	109.116	110.016	95.509	93.612	47.612	23.305
377.5	117.346	110.157	114.141	105.917	106.241	92.488	91.593	46.886	22.045
378	118.021	108.759	112.995	105.559	103.27	91.684	91.211	46.266	21.755
378.5	114.737	108.264	109.967	103.173	103.159	90.543	90.886	45.496	21.326
379	113.449	105.903	108.765	102.534	102.479	89.97	87.579	46.086	21.21
379.5	110.79	103.342	107.189	100.546	100.789	87.881	86.817	45.504	21.208
380	108.249	100.468	103.874	97.193	97.351	84.924	84.648	43.145	19.954
380.5	107.736	98.87	102.146	94.965	95.657	84.844	83.712	42.223	19.918

381	106.401	97.065	100.621	94.113	94.903	83.859	81.895	42.161	19.432
381.5	105.071	95.434	99.305	92.023	91.467	81.81	80.19	40.847	19.924
382	101.714	93.442	95.336	90.708	91.341	79.497	78.286	40.388	17.271
382.5	100.327	92.209	94.001	88.975	89.336	78.657	77.213	39.152	17.823
383	97.264	90.879	94.549	88.486	87.559	77.768	77.657	39.012	17.598
383.5	95.213	89.639	93.085	87.277	87.713	76.406	75.9	38.471	18.427
384	94.402	86.959	89.945	85.457	86.089	75.041	73.822	37.367	16.723
384.5	91.459	86.019	88.164	84.008	82.955	73.713	73.234	36.734	16.525
385	92.093	85.217	87.481	82.495	82.668	73.008	72.506	36.144	16.462
385.5	88.987	83.625	85.271	79.92	80.454	69.984	69.791	35.142	14.176
386	88.496	81.949	84.494	79.956	79.759	71.393	69.495	35.395	15.679
386.5	86.335	79.766	82.269	78.783	78.094	67.861	68.246	34.834	15.64
387	86.318	79.128	82.902	76.879	77.542	68.821	67.766	34.436	15.41
387.5	82.464	78.117	80.353	75.315	74.999	66.42	65.42	33.072	14.126
388	82.476	76.299	79.83	75.311	73.627	65.093	63.306	32.479	14.285
388.5	79.376	74.304	77.123	72.505	72.595	63.011	63.121	32.178	13.857
389	78.2	74.118	76.359	72.102	72.284	62.466	62.658	31.299	13.675
389.5	77.608	71.455	74.218	70.485	70.623	61.241	60.635	31.005	13.542
390	77.854	70.619	73.549	69.128	68.945	60.766	60.309	29.356	13.535
390.5	75.499	70.822	72.323	68.535	67.409	60.413	60.098	29.668	12.866
391	74.325	69.062	70.905	66.424	67.692	59.377	58.839	29.285	12.641
391.5	72.424	67.971	70.556	65.657	65.474	58.542	57.074	29.081	12.186
392	70.253	66.531	67.771	63.36	64.959	56.618	55.53	27.389	12.186
392.5	70.269	64.178	66.814	63.474	63.225	55.885	55.173	27.292	11.856
393	68.367	63.498	66.526	62.257	62.227	54.059	53.762	27.671	11.481
393.5	67.468	62.918	65.334	60.978	61.742	53.359	52.631	26.046	10.199
394	65.568	61.09	64.353	59.713	60.634	53.524	52.614	25.415	10.394
394.5	65.286	60.151	63.092	59.593	58.998	52.499	52.043	25.625	10.752
395	64.699	59.234	62.818	58.797	58.389	51.22	51.126	26.207	10.237
395.5	63.316	57.943	60.997	57.204	56.904	50.708	49.694	24.649	9.792
396	62.971	57.825	61.451	56.143	55.958	49.981	48.705	25.167	10.09
396.5	60.854	56.759	59.34	56.709	55.493	49.15	48.111	24.514	9.778
397	60.1	56.888	58.812	55.132	55.391	48.563	48.024	23.359	9.617

397.5	58.412	54.373	57.02	53.094	53.842	47.272	46.695	23.085	7.874
398	57.962	54.141	55.793	53.707	52.359	46.208	45.51	21.356	8.504
398.5	56.715	52.792	55.871	52.976	51.419	45.68	45.131	21.592	8.145
399	56.278	53.255	55.589	52.62	50.738	44.945	45.268	22.616	8.67
399.5	55.017	50.748	54.016	50.552	50.058	44.585	43.726	22.213	7.583
400	53.136	50.221	51.093	49.122	49.714	42.911	43.196	20.787	7.515

Table 5. Data from Fig. 4 A

H_2O_2 (mM) / Wavelength (nm)	**0**	**10**	**20**	**40**	**80**	**160**
390	0	0	0	0	0	0
390.5	-2.67078	0	-7.1535	-0.63416	0	0
391	-8.7113	0	-13.8696	-4.42464	0	0
391.5	-15.1997	-1.28733	-18.3792	-10.8858	-0.13665	0
392	-18.8966	-6.13629	-16.3805	-17.2479	-4.1305	0.294402
392.5	-14.9122	-13.3637	-2.83499	-19.0551	-11.4002	0.58595
393	1.184498	-19.6681	26.39963	-11.5235	-18.9221	0.216797
393.5	33.75643	-19.8994	75.91503	10.23403	-19.8308	-0.30167
394	87.22903	-8.23011	146.7328	54.84335	-6.44587	-0.89569
394.5	162.455	24.4494	234.4321	125.7591	24.66237	-1.6634
395	255.2035	82.79778	332.1839	218.1295	73.6393	-2.48982
395.5	360.7322	164.7702	437.375	319.2092	138.5015	-3.3134
396	470.3658	261.2772	542.1935	414.3187	212.4939	-4.02251
396.5	571.7312	356.9422	636.2196	489.6218	279.7243	-3.98461
397	653.4156	435.6806	709.7596	533.1643	328.525	-3.19449
397.5	705.0461	485.6018	754.1755	538.0309	349.1492	-2.54007
398	718.7085	498.4015	765.3762	501.6169	339.5584	-1.61842
398.5	695.1024	470.5669	743.4577	434.9056	305.4172	-0.3717
399	637.4878	411.2309	688.8273	353.4636	254.1698	0.967884
399.5	552.2507	334.5948	603.9375	271.0346	195.0075	2.30407
400	453.0814	255.0735	503.0012	197.3849	139.6651	3.41644
400.5	352.7691	184.3525	398.4028	138.4503	97.30975	4.124199
401	260.8641	128.6671	300.2407	96.91274	67.58369	4.764828
401.5	184.7845	88.54525	216.4623	70.5733	49.73435	5.493498
402	128.1353	63.42607	151.2223	55.61828	38.99364	5.701687
402.5	88.87005	49.3366	105.3681	44.54758	31.23846	5.584883
403	65.83942	39.08307	77.54394	35.36551	25.36251	5.295222
403.5	53.03187	30.3829	62.43226	28.33732	20.35293	4.882515

404	44.11145	23.51971	51.81971	22.69748	15.71722	4.491618
404.5	38.17398	18.79169	44.81572	18.79237	13.04996	4.181384
405	34.2779	16.13354	40.23635	16.59746	12.59748	4.057318
405.5	31.83822	15.88656	37.44456	16.29099	12.62059	4.092708
406	30.68856	16.12092	36.19842	16.32782	12.9214	4.255758
406.5	31.15274	16.5406	36.66516	16.46325	13.2981	4.616932
407	31.63781	16.9593	37.11707	16.60223	13.65046	5.083356
407.5	32.01105	17.27575	37.48242	16.70435	14.0007	5.606835
408	32.36562	17.54172	37.72329	16.82414	14.38582	6.194274
408.5	32.67972	17.75459	37.80748	16.93722	14.81398	6.820935
409	32.99887	17.99588	37.80413	17.13395	15.27852	7.461972
409.5	33.3857	18.26745	37.75512	17.40077	15.77063	8.093662
410	33.79451	18.58253	37.76743	17.73802	16.27315	8.701272
410.5	34.2419	18.97061	37.77899	18.10527	16.81677	9.275293
411	34.78116	19.46579	37.80717	18.48487	17.41155	9.869059
411.5	35.4232	20.04456	37.86595	18.89837	18.01176	10.42953
412	36.14241	20.68063	37.99664	19.28674	18.60657	11.0002
412.5	36.98433	21.31858	38.225	19.64141	19.2301	11.62104
413	37.99511	21.9742	38.5167	19.97419	19.87001	12.27929
413.5	39.12254	22.68705	38.86241	20.32733	20.53089	13.00729
414	40.40361	23.44165	39.25673	20.73681	21.25144	13.82084
414.5	41.77846	24.20952	39.74157	21.19138	22.02825	14.69664
415	43.15145	25.01937	40.24937	21.67354	22.85436	15.61252
415.5	44.53922	25.90475	40.82144	22.2178	23.76365	16.62734
416	45.96655	26.89291	41.41279	22.84877	24.75916	17.65441
416.5	47.369	27.98427	42.06567	23.51984	25.79347	18.73167
417	48.82839	29.11156	42.81976	24.22717	26.90332	19.84576
417.5	50.4112	30.3009	43.63864	24.93574	28.05701	20.97493
418	52.09556	31.55901	44.52982	25.66681	29.18828	22.16558
418.5	53.9761	32.91463	45.45553	26.48816	30.31191	23.42051
419	56.03122	34.35082	46.4668	27.385	31.46056	24.71603
419.5	58.23401	35.88259	47.51618	28.33921	32.61818	26.04935
420	60.58466	37.48737	48.61352	29.38476	33.82843	27.45036

420.5	63.13502	39.19683	49.70768	30.50731	35.11897	28.89356
421	65.8117	41.01927	50.87069	31.658	36.49705	30.40456
421.5	68.58416	42.93056	52.14769	32.8457	38.00138	32.02481
422	71.45541	44.88943	53.51775	34.05022	39.6975	33.70227
422.5	74.4006	46.91342	54.9889	35.23924	41.4923	35.48474
423	77.46962	48.98403	56.55382	36.46789	43.36994	37.33105
423.5	80.64815	51.08755	58.26994	37.73942	45.31707	39.23168
424	83.86931	53.28186	60.09582	39.08413	47.26099	41.252
424.5	87.17987	55.52075	62.0159	40.56713	49.21368	43.37099
425	90.6944	57.80847	63.92494	42.1467	51.1201	45.57124
425.5	94.46433	60.17677	65.87264	43.78917	53.00615	47.85466
426	98.37174	62.70756	67.90248	45.50965	54.90589	50.33938
426.5	102.366	65.37977	69.99654	47.31644	56.94716	52.92202
427	106.4234	68.27445	72.13263	49.18546	59.14594	55.62306
427.5	110.5838	71.32485	74.28782	51.10232	61.54421	58.38407
428	114.8138	74.47002	76.53994	53.01901	64.15979	61.16817
428.5	119.0248	77.70067	78.86569	54.98625	66.91249	64.04761
429	123.2083	80.99446	81.29932	57.05645	69.78932	66.98879
429.5	127.4369	84.32775	83.74182	59.19564	72.72684	70.04551
430	131.953	87.64442	86.22101	61.39487	75.68503	73.12184
430.5	136.6714	91.00534	88.76051	63.61052	78.62397	76.41718
431	141.5305	94.36514	91.35159	65.85204	81.55281	79.90516
431.5	146.5811	97.81872	93.99247	68.15334	84.50773	83.52378
432	151.8027	101.4182	96.72671	70.54679	87.56244	87.2072
432.5	157.1756	105.1586	99.58354	73.01782	90.73186	90.99228
433	162.6017	108.9588	102.5271	75.60231	93.93214	94.87664
433.5	167.9803	112.853	105.6793	78.34963	97.21353	98.79584
434	173.2753	116.917	108.9712	81.265	100.6222	102.8284
434.5	178.6137	120.9578	112.3811	84.31142	104.1746	106.8894
435	184.023	125.1292	115.8544	87.4486	107.9281	111.1057
435.5	189.4199	129.4099	119.3817	90.63465	111.8107	115.4798
436	194.8918	133.7626	122.9458	93.81217	115.8715	120.0293
436.5	200.5662	138.2278	126.5382	97.0141	120.083	124.6614

437	206.453	142.8384	130.2255	100.1495	124.4453	129.4144
437.5	212.5925	147.4703	133.9481	103.2861	128.8189	134.3186
438	218.992	152.1099	137.8017	106.4379	133.1608	139.3379
438.5	225.5698	156.8741	141.7722	109.6046	137.4461	144.4117
439	232.268	161.4923	145.9137	112.856	141.7546	149.5459
439.5	239.1017	166.1651	150.0687	116.2152	146.1274	154.7619
440	245.8739	170.9625	154.2432	119.7134	150.5593	160.0465
440.5	252.4255	175.7782	158.3622	123.2674	155.201	165.5042
441	258.9202	180.6737	162.3346	126.9859	160.141	171.0882
441.5	265.4049	185.7477	166.2395	130.7588	165.2613	176.6947
442	271.84	190.9412	170.1064	134.6717	170.4654	182.3921
442.5	278.3452	196.2527	174.0324	138.7452	175.759	188.2452
443	284.9599	201.8408	178.0375	142.862	180.9474	194.1411
443.5	291.641	207.4527	182.229	147.0004	186.1327	200.2003
444	298.5033	213.1088	186.6037	151.1517	191.332	206.4354
444.5	305.558	218.8432	191.1617	155.317	196.4957	212.7241
445	312.4453	224.5435	195.8792	159.3852	201.6986	219.2058
445.5	319.3796	230.1848	200.5839	163.4641	207.1252	225.9357
446	326.4247	235.8247	205.3031	167.4564	212.771	232.7586
446.5	333.405	241.4201	209.9624	171.5183	218.5943	239.7119
447	340.4141	246.9466	214.5482	175.8171	224.6649	246.7653
447.5	347.3772	252.5217	219.0898	180.1891	230.7458	253.7317
448	354.2521	258.1308	223.5762	184.5413	236.9193	260.7599
448.5	361.0442	263.7959	228.0636	188.957	243.2114	267.8857
449	367.8926	269.5043	232.4815	193.5171	249.5237	275.0351
449.5	374.5816	275.2006	237.0295	198.1023	255.7727	282.2249
450	381.1064	280.8466	241.6561	202.7737	262.1409	289.5168
450.5	387.6669	286.5158	246.4557	207.3744	268.6257	296.9332
451	394.2799	292.0661	251.3713	212.0715	275.1393	304.5327
451.5	400.9309	297.5605	256.3328	217.0718	281.8294	312.2147
452	407.6471	303.0729	261.3435	222.2177	288.4259	319.8464
452.5	414.3537	308.5313	266.2759	227.2019	294.9081	327.433
453	420.9511	314.0583	271.1426	232.0508	301.282	335.0489

453.5	427.4748	319.4654	275.7535	236.7225	307.4788	342.4632
454	433.8381	324.8677	280.2823	241.1423	313.3626	349.7076
454.5	439.8257	330.1961	284.5611	245.4103	319.0489	356.7489
455	445.5613	335.5052	288.8952	249.4231	324.8077	363.7766
455.5	451.1512	340.6741	293.3209	253.3537	330.5924	370.8683
456	456.5278	345.8238	297.737	257.5198	336.595	378.1176
456.5	461.8736	351.0565	302.2591	261.9614	342.735	385.5571
457	467.3116	356.1345	306.7562	266.5113	349.0877	393.2134
457.5	472.6479	361.1854	311.2535	271.1736	355.6436	401.0915
458	477.881	366.0691	315.5786	275.9257	362.2759	408.9071
458.5	483.143	370.9358	319.8197	280.5969	368.7757	416.5227
459	488.3472	375.7421	323.7647	285.2593	375.1319	423.8557
459.5	493.422	380.4071	327.4945	289.6883	381.3145	431.0295
460	498.3551	384.778	331.2414	293.6187	387.2458	437.9262
460.5	502.8623	388.9929	334.9344	297.3272	392.928	444.4769
461	507.1309	393.3199	338.7042	300.8174	398.3469	450.761
461.5	511.2952	397.6061	342.4334	304.1092	403.5656	456.9424
462	515.167	401.8093	346.1392	307.2041	408.6504	463.1382
462.5	518.8038	405.8639	349.8423	310.2665	413.6212	469.1837
463	522.3356	409.6793	353.6183	313.3535	418.4143	474.9936
463.5	525.8227	413.3201	357.3502	316.7423	423.0585	480.5782
464	529.2305	416.6927	360.7941	320.4042	427.4605	486.1897
464.5	532.5629	419.6962	364.1209	323.9716	431.7851	491.8429
465	535.6333	422.363	367.1423	327.6166	436.0308	497.5096
465.5	538.5024	424.9507	370.0475	331.1757	440.1811	503.076
466	541.1701	427.5997	372.6889	334.5227	444.313	508.6153
466.5	543.5591	430.3094	375.0751	337.616	448.6163	514.0123
467	545.6205	433.1544	377.2354	340.5139	453.1532	519.2323
467.5	547.3844	435.9191	379.3699	343.168	457.6527	524.1463
468	549.1025	438.6275	381.6468	345.8548	462.0948	528.6836
468.5	550.7951	441.1588	383.817	348.5914	466.1936	532.9343
469	552.5579	443.3925	385.958	351.1663	469.9461	536.9573
469.5	554.1401	445.3191	387.901	353.857	473.3437	540.8484

470	555.6437	446.8452	389.7969	356.5916	476.1072	544.4993
470.5	556.8582	448.1787	391.4383	359.141	478.262	548.0677
471	557.8436	449.409	392.8206	361.5934	480.2079	551.5455
471.5	558.4974	450.7203	393.864	363.8957	482.4833	554.8372
472	558.838	452.014	394.667	365.9304	484.9399	557.8518
472.5	558.9499	453.2893	395.4802	367.7371	487.5787	560.6427
473	558.9286	454.4217	396.3219	369.146	490.3689	563.2305
473.5	558.9624	455.3957	397.14	370.0749	493.1843	565.512
474	558.853	456.3551	397.9147	370.879	496.1361	567.4899
474.5	558.6783	457.1349	398.8275	371.5492	498.6529	569.2565
475	558.421	457.7431	399.5506	372.0249	500.4588	571.0103
475.5	558.3224	458.3066	400.1273	372.5187	501.4938	572.9195
476	558.215	458.6677	400.5571	373.2069	502.2987	574.9245
476.5	557.9056	458.8881	400.7169	374.1281	502.9189	576.9736
477	557.2296	459.0201	400.763	375.1787	503.2581	579.1219
477.5	556.3651	458.9062	400.8658	376.252	503.7075	581.3546
478	555.4588	458.5799	400.8328	377.1986	504.2623	583.4961
478.5	554.3026	458.2559	400.8772	378.0744	505.2398	585.3302
479	552.8196	457.8323	401.1251	378.664	506.3611	586.728
479.5	551.204	457.3276	401.3516	379.1605	507.4603	587.696
480	549.8006	456.9111	401.4197	379.394	508.2789	588.5207
480.5	548.4273	456.3038	401.3514	379.4064	509.0067	589.2028
481	547.1874	455.7196	401.0441	379.358	509.6557	589.9343
481.5	545.7806	455.2514	400.522	379.1039	509.9727	590.6364
482	544.3	454.7655	400.0749	378.6766	510.3082	591.3279
482.5	542.8701	454.2266	399.2725	378.1153	510.6791	591.9577
483	541.3523	453.6819	398.3857	377.427	510.925	592.4144
483.5	539.5456	453.0604	397.5941	376.5522	511.0776	592.6196
484	537.5149	452.2222	396.8933	375.8674	511.0846	592.2746
484.5	535.5736	451.3871	396.1032	375.2403	510.8442	591.6402
485	533.6448	450.1896	395.2974	374.6962	510.2835	590.7692
485.5	531.9468	448.7528	394.5346	374.3236	509.4656	590.1234
486	530.2594	447.1944	393.6591	373.9737	508.2762	589.5794

486.5	528.5487	445.5171	392.9282	373.5216	507.0915	589.1348
487	526.8479	443.798	392.1601	372.9603	506.1636	588.6852
487.5	525.0339	442.1367	391.3389	371.9745	505.1568	588.3057
488	522.901	440.5507	390.4995	370.5041	504.1655	588.0069
488.5	520.3756	438.9128	389.6504	368.992	503.1748	587.3112
489	517.6072	437.3755	388.622	367.5595	502.2402	586.2384
489.5	514.4559	435.7869	387.3621	366.2624	501.3805	584.5452
490	511.2147	434.094	386.0558	365.2731	500.3749	582.7702

Table 6. Data from Fig. 4 B

Sample	Area	Percent (%)
0 mM	9829.083	9.646
5 mM	10718.98	10.519
10 mM	14890.81	14.613
20 mM	18643.23	18.295
40 mM	16968.23	16.652
80 mM	12834.76	12.595
100 mM	9513.569	9.336
160 mM	8502.497	8.344

Table 7. Data from Fig. 5 B

H_2O_2 (mM) / Wavelength (nm)	0 mM-50 °C	10 mM-50 °C	25 mM-50 °C	50 mM-50 °C	100 mM-50 °C	0 mM-70 °C	10 mM-70 °C	25 mM-70 °C	50 mM-70 °C	100 mM-70 °C
300	458.3141	341.206	309.575	366.275	369.179	509.2384	420.665	380.742	363.792	520.308
300.5	452.8517	335.8548	304.4707	361.8229	361.9075	505.9114	415.4488	376.9466	357.2566	511.4283
301	447.3023	330.8217	299.7907	357.4785	355.2363	502.5625	410.4261	373.2755	351.4362	502.3965
301.5	442.5467	327.0084	295.9548	353.8251	349.9153	499.2225	406.3423	369.9863	347.0613	494.3289
302	438.7566	324.1085	293.2065	350.7892	346.3332	496.2329	403.083	367.2847	344.0003	487.7947
302.5	436.3291	322.2851	291.66	348.5287	344.2425	493.6353	400.6936	364.7404	342.2866	482.2478
303	434.7029	321.4482	291.1837	346.8962	343.6348	491.6064	398.988	363.1382	341.8983	478.723
303.5	433.4922	321.4978	291.2101	345.7778	343.9053	489.7629	397.8436	361.8541	342.3513	476.1914
304	432.407	321.8475	291.5044	344.914	345.0921	488.8542	396.9107	360.9003	343.0077	474.3436
304.5	432.1942	322.3977	292.003	344.5021	346.4903	489.0779	396.3833	360.6102	343.5153	473.2364
305	432.573	323.3032	292.6386	344.6108	347.8334	489.9057	395.9868	360.7115	343.8072	472.6614
305.5	433.4816	324.1738	293.3238	344.721	348.8465	491.2672	395.7325	361.1821	343.9519	471.366
306	434.6919	325.3548	293.9452	345.3241	349.9648	493.0814	396.1666	361.9962	344.4265	469.7552
306.5	435.9671	326.5731	294.56	346.1163	351.207	495.2369	396.9449	363.1965	344.9577	467.912
307	438.06	328.0132	295.4913	347.3765	352.6297	497.5416	398.0471	364.4708	345.726	465.8581
307.5	440.6146	329.6753	296.9274	348.8966	354.151	500.4142	399.5035	366.2802	346.8818	464.5837
308	442.9292	331.4735	298.3883	350.6602	355.8187	503.2091	401.254	368.1506	348.2259	463.8314
308.5	444.7262	333.5205	299.8655	352.0937	357.8523	506.183	403.3515	369.8848	349.8168	463.516
309	446.2642	335.4784	301.4855	353.5064	360.0836	509.584	405.5706	371.5916	351.3248	463.6989
309.5	447.405	337.7797	303.2461	354.8362	362.3423	512.8815	407.7635	373.3677	352.7474	464.2901
310	448.775	340.29	305.4559	355.9588	364.6044	516.1765	409.9406	375.2979	354.1489	464.892
310.5	450.1035	342.9442	307.7476	357.1866	366.7565	519.6592	412.3908	377.2755	355.6496	465.6114
311	451.3496	345.6795	310.1085	358.4228	368.6664	523.7284	414.7953	379.2017	357.1486	466.0816
311.5	453.0188	348.5388	312.7758	359.8584	370.7552	528.0426	417.3029	381.1216	358.9109	466.3239
312	455.269	351.4801	315.9988	361.5287	372.6609	532.6321	419.7967	383.3926	360.9866	466.7526
312.5	457.9274	354.735	319.2202	363.8259	374.6781	537.5592	422.2951	385.6682	363.3249	467.5129
313	461.0301	358.617	322.7567	366.0283	376.9155	543.1321	425.0848	388.0293	366.0966	468.7775
313.5	464.3746	362.5247	326.2235	368.6184	379.4376	549.415	427.8403	390.456	369.0738	470.7434

314	467.8737	366.8476	329.6795	371.0594	382.3103	556.1043	430.8014	393.1861	372.0895	472.5293
314.5	471.9155	371.6254	333.2368	373.513	385.8245	562.9655	433.9515	396.0735	374.8845	474.4276
315	476.3354	376.3658	336.7422	376.1266	389.7186	569.6698	437.703	399.0751	377.5405	476.2242
315.5	480.9588	381.1156	340.1171	378.8299	393.4095	576.1364	441.4203	402.2569	380.0964	477.6067
316	485.6086	385.9075	343.6231	381.7923	397.2279	582.5264	445.4412	405.5028	382.9412	478.8989
316.5	490.2896	390.3748	347.3945	384.9332	400.94	587.9361	449.5685	409.133	385.9827	480.0241
317	495.3087	394.802	351.1275	388.4729	404.8192	593.1111	453.8106	412.9904	389.4111	481.2257
317.5	500.5161	399.3692	355.3194	392.0928	408.7317	598.5071	458.2756	416.9329	393.235	482.5975
318	505.5536	403.6135	359.654	396.4474	412.4225	604.1525	462.8038	420.8891	397.4807	484.7842
318.5	510.8001	408.1221	364.3654	400.7655	415.9287	610.0249	467.4651	425.0271	401.9622	487.274
319	516.1473	413.0058	369.459	405.0031	419.5371	616.3145	472.0835	429.4115	406.3908	490.1929
319.5	522.0904	417.8467	374.7636	409.1003	423.3919	623.2374	477.0413	433.6685	410.716	493.2407
320	528.6641	422.6602	380.0988	412.9983	427.0355	630.5882	481.9047	438.0423	414.7656	496.073
320.5	535.3774	427.7977	385.702	416.715	430.6294	638.562	486.6827	442.1217	418.5748	499.2121
321	541.8993	433.1634	391.4951	420.3346	434.3974	646.6127	491.5732	446.2632	422.0632	502.2226
321.5	548.7545	438.8254	396.9803	423.905	438.4721	655.0243	496.5205	450.5757	425.4755	505.1893
322	555.6787	444.6747	402.3222	427.1315	443.1508	663.7583	501.6312	454.8193	429.1	507.8119
322.5	562.0172	450.3521	407.2222	430.8186	448.1135	672.6383	506.8778	459.0925	433.0736	510.5233
323	568.3329	456.5661	412.2208	434.7771	453.5829	682.0147	512.3947	463.3931	437.4882	513.5645
323.5	574.1605	463.064	417.1913	439.0699	459.1063	691.1429	518.0111	467.7799	442.0197	517.0363
324	580.191	469.6024	422.1399	443.6648	464.979	700.6046	523.9156	472.2069	446.8594	520.8503
324.5	586.2555	475.8337	426.9641	448.5506	470.817	710.0686	530.2744	476.8461	451.7856	524.4364
325	592.4732	482.0069	432.0795	453.7107	476.0927	719.0176	536.5912	481.4175	456.6905	528.1041
325.5	599.0169	488.3694	437.8692	458.8688	480.818	727.6416	542.9201	486.3602	461.5665	531.7462
326	605.9572	494.7617	443.974	464.1697	485.1077	736.0299	548.861	491.9879	466.2387	535.8103
326.5	613.4298	501.2239	450.0923	469.3787	489.4695	744.3071	554.4013	498.0715	470.769	539.7929
327	620.7566	507.4868	456.1317	474.6525	493.4436	752.4681	559.429	504.4347	475.1379	543.1065
327.5	627.9	514.1167	462.1958	479.7514	497.4513	760.9416	564.1588	510.7198	479.6094	546.3638
328	634.178	520.8327	468.2853	484.6129	501.1665	769.3397	568.6493	516.7424	483.8056	549.6536
328.5	640.0097	527.5801	474.0884	489.1846	504.988	777.8893	572.82	522.3449	487.8408	552.9527
329	645.1389	533.9678	479.2705	493.6838	508.9651	786.8369	577.4361	527.36	491.5753	556.1626
329.5	649.5778	540.0615	484.0643	497.9521	512.8115	795.5265	582.3171	531.5312	495.2363	558.8303
330	654.0201	545.9197	489.0108	501.604	516.589	804.2436	587.6811	535.2173	499.0367	561.1576

330.5	658.2089	551.2206	493.9919	504.8776	519.9404	812.4507	593.044	538.6757	502.7674	563.6788
331	662.889	556.0365	498.7987	508.452	523.5992	820.3196	598.556	542.113	506.4889	566.5721
331.5	668.2074	560.365	503.3694	512.1099	526.9353	827.3999	603.7328	545.8334	509.863	569.1274
332	674.032	564.3372	507.7875	515.7002	530.1763	834.2439	608.3925	549.5458	513.4094	571.4885
332.5	679.7974	568.5382	512.5041	519.1178	533.2606	841.1243	612.7221	553.3214	517.0624	573.9399
333	685.6633	572.8952	517.246	522.3493	536.5062	847.7264	616.2804	557.1345	520.6084	576.611
333.5	691.6087	577.0957	521.8089	525.4655	540.2333	854.3357	619.3473	560.8272	523.6831	579.6556
334	696.9754	581.6813	525.9687	528.6413	543.9651	860.8984	622.3386	563.9108	526.3605	582.5186
334.5	702.1833	586.4962	529.9151	531.4366	547.7938	867.9784	625.4581	566.7495	528.9247	584.9175
335	706.4753	591.2818	533.9331	533.6744	550.9262	874.8055	628.7983	569.716	531.488	587.0587
335.5	710.2344	596.1241	537.8445	536.0959	554.0013	881.7702	632.2446	572.8001	534.0309	589.2338
336	714.1158	600.8231	541.4536	538.6323	556.9097	888.1188	635.8708	576.0872	536.1811	591.0147
336.5	718.3783	605.0302	544.6321	541.1959	559.1047	893.9489	639.3913	579.0669	538.186	592.3871
337	722.1496	609.2925	547.8813	543.7131	560.756	899.8292	643.0505	582.1353	540.2711	593.3292
337.5	725.6829	613.1232	550.9922	546.2607	562.0238	905.5088	646.5231	585.065	542.7345	594.1256
338	729.5436	616.3859	554.1623	548.7047	563.3805	910.7551	649.9471	587.8426	545.2402	595.1935
338.5	733.2112	619.3876	556.9892	551.2305	564.4758	915.2107	653.0912	589.8829	547.5587	596.4292
339	736.9397	622.1549	559.4007	553.6467	565.8725	919.631	655.9835	591.268	549.7247	597.5662
339.5	740.1997	624.7164	561.7146	555.7532	567.0303	923.5413	658.9447	592.3806	551.8091	598.3331
340	742.8932	627.1195	563.8133	557.4781	567.9736	927.2288	661.6707	593.2736	553.8393	598.7472
340.5	745.3436	629.156	565.6611	559.0551	569.121	929.9925	664.1831	594.0044	555.6613	598.8452
341	747.9289	630.9516	567.0283	560.5106	569.9473	932.0126	666.2758	594.5955	556.8675	599.0441
341.5	749.7312	633.0564	568.3061	561.7557	570.4221	933.6705	668.1188	595.3978	557.5001	599.0165
342	751.3102	634.8526	569.6662	562.8942	570.8827	935.6776	669.5665	596.7519	558.0107	598.5405
342.5	753.4197	636.6309	571.7786	563.8252	571.2254	937.9709	670.7462	598.5209	558.6034	598.3563
343	755.2832	638.3406	574.1591	564.6719	571.4973	939.9524	671.7674	600.3971	559.0295	598.9014
343.5	757.33	640.393	576.7767	565.5666	572.3006	942.3208	672.6125	602.1376	559.3827	599.9143
344	759.2475	642.5194	579.48	566.4361	573.4415	944.6527	673.5205	603.9759	559.9373	601.0111
344.5	761.1357	644.8795	582.1212	566.7873	574.5058	946.9409	674.3572	605.7728	560.473	602.1304
345	762.9131	646.9275	584.4873	566.889	576.1913	949.3735	675.25	606.814	561.1625	602.672
345.5	764.7609	649.0481	585.9669	566.9256	578.2042	951.6608	676.3713	607.4472	561.7573	603.082
346	766.0468	651.4676	586.5747	566.9466	580.036	953.6898	677.4412	607.8316	562.1058	603.2796
346.5	766.8995	653.3753	586.6316	566.7312	582.0386	955.5421	678.4976	608.3657	562.2579	602.4694

347	767.889	655.1379	586.7037	566.6351	583.6934	957.3228	679.275	608.6159	562.4185	601.2879
347.5	768.4504	656.384	586.5155	566.4743	584.9512	959.4766	680.0273	608.8365	562.3893	600.0682
348	768.8224	657.6172	586.7676	566.6606	585.8973	961.4316	680.6278	608.835	562.3631	599.2078
348.5	768.7306	658.4443	587.2336	566.9966	586.7374	963.1503	681.0783	608.7404	562.3988	598.662
349	769.0282	659.1517	588.0195	567.1886	587.2636	964.1529	681.4131	608.7595	562.5394	598.3449
349.5	769.6034	659.2839	589.2508	567.3822	587.6607	965.0985	681.4065	608.4701	562.8124	597.9755
350	770.2231	659.3106	590.3222	567.3938	588.3139	966.0743	681.5341	608.3167	562.938	597.4408
350.5	771.2492	659.5292	590.9818	567.5211	588.7126	967.0562	681.4683	608.0685	562.9229	597.1256
351	771.99	659.7147	591.3869	567.5192	588.8129	967.1149	681.515	608.1338	562.826	596.6468
351.5	772.7697	660.107	591.7367	567.6616	588.4696	966.6011	681.2258	608.0469	562.5866	595.8553
352	773.3881	660.4071	591.7013	567.7273	587.6774	966.4367	680.8145	608.1592	562.1573	594.6457
352.5	773.686	660.8317	591.8687	568.0802	586.5684	966.0483	679.8853	608.0538	561.5374	593.7691
353	773.1084	661.3418	591.8535	568.3929	585.3272	965.3761	678.7739	607.5034	560.8367	592.9767
353.5	772.4001	661.9628	591.9545	568.4783	584.1173	963.8266	677.7175	606.582	560.4154	592.1379
354	771.3538	662.3145	591.9765	568.5257	582.9251	961.7492	676.6339	605.5011	560.197	591.3058
354.5	769.6429	662.55	591.7616	568.1267	582.0282	960.1671	675.9163	604.7175	559.9884	590.3754
355	768.5021	662.5643	591.5082	567.5355	581.635	959.406	675.1713	604.1217	559.8467	589.1293
355.5	767.3377	662.1399	591.3196	566.7937	581.1203	958.0227	674.7043	603.6172	559.9553	587.92
356	766.208	661.2611	591.1533	565.9932	580.476	956.609	674.4356	603.0938	559.7093	586.6714
356.5	765.3414	659.8537	590.3913	564.7181	579.4679	955.373	674.234	602.8619	559.0864	585.1637
357	764.6484	657.8935	589.4887	563.1532	577.9445	954.2665	673.6584	602.7528	558.129	584.3623
357.5	763.7321	655.725	588.1859	561.63	576.0669	953.0162	672.7752	602.0798	556.5816	584.1912
358	762.7853	653.6708	586.6968	559.9653	574.1738	951.1067	671.7807	600.9206	554.9584	583.9919
358.5	761.6857	651.5564	584.7406	558.6103	572.2532	947.9439	670.3283	599.3736	553.3429	583.7437
359	759.8456	649.9068	582.5951	557.045	570.4984	944.6269	668.7289	597.5761	551.4295	583.8841
359.5	758.1998	648.8923	580.6075	555.5075	569.3548	941.6026	666.766	595.5897	549.5309	583.5736
360	756.0584	648.173	578.864	554.4107	568.6248	937.9774	664.5399	593.2491	548.1995	582.9595
360.5	753.6033	647.5641	577.5682	553.7057	567.9909	934.7573	662.2063	590.6971	547.0254	581.5841
361	750.9848	646.8913	576.3237	552.9975	567.0563	931.5356	659.8274	588.2386	545.8429	579.2256
361.5	748.4292	645.9613	575.2658	551.9381	565.9253	928.951	657.584	585.939	544.6661	576.9488
362	746.3599	644.6711	574.0236	551.0473	564.2601	927.006	655.3842	583.5238	543.3442	574.6077
362.5	744.416	643.1871	572.7001	550.125	562.053	925.3341	653.456	581.6181	541.6723	572.1009
363	742.5655	641.1303	571.0171	548.9728	559.2764	922.9468	651.2736	580.1226	539.8242	569.5912

363.5	740.4521	638.8613	569.1672	547.1064	556.1466	919.9071	648.8955	578.8073	537.4594	567.9581
364	738.7692	636.786	567.2236	544.7483	553.3973	916.2108	646.5586	577.5845	534.9066	566.6882
364.5	736.7619	634.7146	565.3762	542.2241	551.1999	911.4331	644.0864	576.2956	532.4464	565.8649
365	734.4329	632.488	563.7604	539.832	549.058	906.1214	641.3678	574.8832	530.2102	565.086
365.5	731.6249	629.8563	562.4419	537.4519	547.2644	900.2483	638.3935	573.5502	528.0265	564.1597
366	728.3093	626.9365	561.3887	534.9158	545.8639	894.6533	635.6021	572.0331	526.2259	563.325
366.5	725.2107	623.884	560.5209	532.5306	544.7079	889.7022	632.9995	569.986	524.7511	562.1243
367	721.7672	621.2866	559.5457	530.6562	543.7438	885.4889	631.1632	568.214	523.1906	560.1095
367.5	718.066	618.562	558.1318	528.9254	542.0927	881.8613	629.3567	566.7561	521.6732	557.7968
368	714.0978	615.7959	556.5926	526.9585	539.8612	878.6761	627.3482	565.4723	520.212	555.6917
368.5	710.5836	613.2283	554.6106	524.8977	537.5699	875.7607	625.4564	564.0612	518.2981	553.4444
369	707.1652	610.7543	552.2888	522.7178	535.553	872.886	623.1948	562.7164	515.8407	551.4334
369.5	703.981	608.6629	549.5141	520.3647	533.1035	869.5487	620.3516	561.0025	513.2169	549.9075
370	700.9374	606.8638	546.9448	517.8544	530.5701	865.6007	616.8498	559.2267	510.5081	548.4783
370.5	697.709	604.7972	544.2127	515.43	528.0491	861.543	613.0774	556.9781	507.6859	547.3075
371	694.7217	602.2772	541.4577	512.8119	525.315	857.3541	609.3419	553.8146	505.2989	546.0474
371.5	692.0398	599.9239	538.8324	510.3817	522.9862	853.0022	606.1476	550.3083	502.886	544.4356
372	688.9865	597.3311	536.0528	507.729	520.4653	848.2533	603.2227	546.4911	500.6408	542.9043
372.5	685.7904	594.5626	533.3579	505.0965	517.5404	843.4685	600.4599	542.6553	498.9012	541.6687
373	682.4115	591.6643	530.6348	502.4997	514.4327	838.3565	598.2458	538.8453	497.2923	540.7456
373.5	678.9005	588.4154	527.995	500.0969	511.7753	833.3021	596.0322	535.4423	495.4725	539.5171
374	675.4331	585.1788	525.1549	497.6206	508.9702	828.031	593.5825	532.4298	493.4005	538.4838
374.5	672.1671	582.3671	522.6463	495.1019	506.4734	822.3827	590.5974	530.3813	491.5416	537.3857
375	668.3574	579.391	519.7824	493.1686	503.9844	816.6053	587.1453	528.9053	489.5301	536.2431
375.5	664.2522	576.2755	516.8108	491.099	501.3137	811.2692	583.54	527.1977	487.6929	534.7999
376	660.5952	573.3924	513.9785	489.2629	498.8759	806.8066	579.962	525.2914	485.7339	532.9801
376.5	656.638	570.3635	511.0517	487.0954	496.5264	802.7483	576.2938	523.1021	483.4684	530.9952
377	652.6955	567.2237	507.9508	484.7269	494.0383	799.0354	572.7122	520.4507	481.3045	528.9842
377.5	648.629	564.179	504.8376	482.0439	491.0132	794.9716	569.5647	517.293	478.9896	527.3978
378	644.7053	560.5193	501.827	479.3545	488.0362	790.8953	566.6247	513.8999	476.1716	525.4692
378.5	640.885	556.7122	498.8858	476.2537	484.9062	786.683	563.8559	510.4205	472.6857	523.337
379	637.4413	553.288	496.2988	473.1491	481.7911	782.1023	561.0137	507.3265	469.0164	521.1647
379.5	633.6283	550.1026	493.5765	470.5115	478.7147	776.4355	557.8045	504.6351	465.2129	519.0811

380	629.0725	546.8732	490.8529	467.5551	475.487	770.1195	554.5359	502.0114	461.6221	517.1527
380.5	625.2727	543.8869	488.0936	464.7118	472.3448	764.1364	551.2112	499.4036	458.3911	515.0499
381	621.5076	540.7719	485.4491	461.8886	469.3402	758.3429	547.733	496.7915	455.3435	512.8863
381.5	618.0373	537.5554	482.5592	458.9345	466.4646	753.178	544.1381	493.8235	452.6172	510.5623
382	614.8001	534.4829	479.2525	455.9128	463.2877	747.7714	540.4533	490.6228	450.2014	509.0418
382.5	611.9144	530.9561	475.8735	452.8082	460.2617	742.4971	536.7174	487.0792	447.9821	507.8321
383	609.15	527.3047	472.6476	449.3389	457.415	737.7953	532.7837	483.3833	445.6714	506.17
383.5	606.7909	523.9096	469.4568	445.9871	454.8205	733.8074	528.9687	480.1148	443.2117	504.4494
384	604.2022	520.5875	466.2295	442.8809	452.2681	729.4732	525.3653	476.9249	440.5779	502.7411
384.5	600.3879	517.3566	463.0041	439.5935	449.6988	724.6868	522.0149	474.274	437.69	501.303
385	596.4929	514.2217	459.4014	436.6545	447.1346	719.894	518.855	471.8412	434.8097	499.6444
385.5	592.0443	511.0375	456.0792	434.1856	444.5114	715.1606	516.161	469.784	431.9059	497.7152
386	587.3472	507.8551	452.9092	431.949	441.8528	710.9225	513.781	467.4703	429.2367	495.4345
386.5	582.5536	504.9851	449.6765	430.2184	438.9934	706.1357	511.4155	465.1384	426.5576	493.1114
387	577.6514	502.0026	446.6405	428.4803	436.1311	700.6772	509.1	462.6213	423.7531	491.0531
387.5	572.9682	499.0564	443.9556	426.4243	433.1082	694.8055	506.2857	459.754	421.1799	488.5293
388	568.9058	496.3095	441.3572	424.5463	429.9674	688.9625	502.9327	456.8877	418.6311	486.2393
388.5	565.8163	493.6069	439.1921	422.3412	426.9095	682.9574	499.2609	453.5337	416.0853	484.189
389	562.4377	490.7623	437.3177	419.6624	423.8741	676.4707	495.3404	450.3361	413.4533	482.7306
389.5	559.4663	487.8412	435.1131	416.8997	420.9873	670.3806	491.1949	447.0495	410.8139	481.4074
390	556.3024	484.6413	432.9564	413.8796	418.0577	664.6175	487.0358	443.8675	408.2248	480.0369
390.5	553.265	480.8877	430.6255	410.7989	415.008	659.9361	483.0156	440.4681	405.8367	478.4608
391	550.1915	476.8088	427.7966	408.0205	412.0625	655.5264	479.0618	437.4028	403.5684	476.5106
391.5	546.3063	472.6051	424.5269	405.163	409.6062	651.4461	475.7077	434.5115	401.1451	474.442
392	541.812	468.5286	420.9373	402.2506	407.0941	647.2384	472.3571	431.4596	398.9283	471.5549
392.5	536.9859	464.9638	417.1922	399.9461	404.8117	643.5644	468.7231	428.3778	396.7908	468.6321
393	532.2818	461.5623	413.6932	397.6132	402.6922	640.1585	465.2771	425.0687	394.3136	465.741
393.5	527.4498	458.2608	410.3852	395.0102	400.7387	636.1681	461.9784	422.0588	391.4481	463.5003
394	522.8599	455.0803	406.9822	392.4936	398.8246	632.0282	458.5724	419.0864	388.311	461.5406
394.5	518.4387	452.3812	403.8189	389.6983	396.8391	627.7402	455.2908	416.1479	385.0932	460.0461
395	514.5359	449.4628	400.9009	387.1413	394.4847	623.4338	452.056	413.1567	381.9791	458.998
395.5	511.3025	446.729	398.2875	384.833	391.8383	618.5024	448.82	410.548	379.0397	457.9582
396	508.1297	443.8316	395.9153	382.4283	389.2405	613.2901	446.0198	408.0878	376.2548	457.1629

396.5	505.1284	440.833	393.4229	379.8876	386.3452	607.3007	443.2801	405.7196	374.1348	455.9057
397	501.9537	438.064	390.9958	377.603	383.5592	601.4615	440.1104	403.0043	372.3123	454.5438
397.5	498.6245	435.1007	388.8225	375.5564	380.7882	595.9705	436.9022	400.1871	370.4643	452.683
398	495.0517	431.8099	386.4219	373.5349	378.15	590.2148	433.6385	397.328	368.3291	450.9612
398.5	491.326	428.1527	383.7173	371.3225	375.5477	584.7525	430.3579	394.433	365.9188	449.1273
399	487.3317	424.7324	380.7988	368.7439	373.1205	579.9112	427.0523	391.3512	363.3997	447.6246
399.5	483.1241	420.905	377.8664	366.2156	370.5563	575.5438	423.7427	388.2612	360.836	446.0955
400	479.1459	417.3322	374.8957	363.7925	367.9855	571.267	420.5267	385.6222	358.0797	444.2084

Table 8. Data from Fig. 6 A

H_2O_2 (mM) / Wavelength (nm)	0 mM-50 °C	10 mM-50 °C	25 mM-50 °C	50 mM-50 °C	100 mM-50 °C	0 mM-70 °C	10 mM-70 °C	25 mM-70 °C	50 mM-70 °C	100 mM-70 °C
400	425.273	422.077	379.05	349.161	284.295	419.798	374.158	361.773	294.701	287.594
400.5	432.0943	423.6235	382.8228	355.699	286.5388	422.4227	381.3404	367.0393	296.4122	287.3262
401	438.3847	426.1525	386.9782	361.6753	288.8617	425.7924	388.3715	371.9882	298.3602	287.4249
401.5	444.1019	429.5184	390.9215	367.0768	291.1269	429.1595	394.7232	376.6571	300.6035	287.9138
402	449.5403	432.9451	394.6847	372.0953	293.4255	432.6604	400.9825	381.3178	303.1357	289.525
402.5	454.8186	437.3934	398.565	376.5383	296.0273	437.2826	406.458	385.6547	306.0247	292.0519
403	459.8492	442.3875	402.9156	380.4765	298.4108	442.1093	411.4642	389.2504	309.3837	294.6841
403.5	465.0315	448.3889	408.1563	383.8379	300.8202	447.588	415.9847	392.2581	313.0334	297.3094
404	470.0347	454.7964	413.9835	387.1045	302.9488	454.0651	420.2767	395.2004	316.5407	299.894
404.5	475.9495	460.8943	420.0941	390.5351	304.9644	460.6492	424.3535	398.6151	319.8689	302.11
405	481.8166	466.4325	425.3975	393.839	307.3182	466.6241	428.3625	401.975	322.9301	303.8448
405.5	487.9584	472.307	430.6157	397.2089	310.0432	472.8916	432.6155	405.4924	325.9258	304.9902
406	494.0037	478.5119	435.1889	400.253	312.6923	478.786	436.4061	409.2163	328.2912	305.5225
406.5	499.9817	484.0745	438.9371	403.2881	315.4739	483.8474	440.7669	413.6107	330.4346	306.2689
407	505.7687	489.3675	442.1081	406.6926	318.669	488.6231	444.9201	418.0576	332.1739	307.6002
407.5	511.1447	494.2124	444.7645	410.2051	321.8663	493.0214	448.7008	422.1024	334.0594	308.8324
408	516.1204	499.0983	447.1866	413.6315	325.0496	496.7331	452.5396	425.2474	335.9007	310.3036
408.5	520.4763	504.6211	450.1991	417.2058	327.7032	500.3888	456.4649	428.0461	337.5967	312.4845
409	524.9755	509.8208	454.2361	421.076	330.2485	503.9953	460.046	431.3738	339.3889	315.1697
409.5	529.1611	514.4309	458.4938	425.0321	332.9547	507.387	463.2313	434.5901	341.0909	318.2383
410	533.3181	519.2241	463.116	429.3377	335.8249	511.4518	466.4376	437.6481	343.1751	321.165
410.5	537.2244	523.9708	467.9297	433.1878	338.1977	515.9625	469.3193	440.418	344.87	323.5884
411	541.0775	527.9332	472.8155	436.7932	340.2049	520.8842	472.6541	443.627	346.7704	325.9785
411.5	544.9301	531.4163	477.4887	440.4555	342.1637	526.0375	476.1957	446.7828	348.366	328.4011
412	549.0337	534.5288	481.9757	443.3747	343.8597	531.703	479.5135	449.9507	350.3414	330.1943
412.5	553.0998	537.4103	485.848	445.8566	345.4456	537.3018	482.9337	452.4967	352.3337	331.6944
413	557.0591	540.9941	489.8235	448.1894	346.7979	542.4758	487.0032	454.7011	354.1076	333.347
413.5	560.78	544.6986	493.7223	450.5218	347.8908	546.6274	491.3893	457.4224	356.0373	334.9634

414	564.8325	548.6789	497.1962	453.1837	349.1195	549.9466	495.7643	460.5199	358.1605	336.6374
414.5	568.894	553.1718	499.9064	456.3328	350.881	552.9678	500.1403	463.6725	360.8231	338.2472
415	572.732	557.5111	502.5526	459.4855	352.8005	555.7964	504.455	466.4473	363.2634	339.8094
415.5	576.5764	561.4227	505.3193	463.0372	354.8049	558.8165	508.2469	469.1226	365.7228	341.5558
416	580.4471	565.4139	507.8609	466.9077	357.0313	562.0405	511.8778	471.2995	367.9418	343.4464
416.5	584.6219	568.7522	510.4145	469.9958	359.2893	565.4742	514.7004	473.0932	370.3032	344.9207
417	588.5967	571.4702	512.3719	472.7571	361.1786	569.2639	516.7687	474.1614	372.6949	346.2149
417.5	592.2469	574.0142	514.3233	474.8518	362.9062	572.9204	518.5287	474.609	374.4207	347.539
418	595.2231	576.1727	516.1823	476.4466	364.1877	575.7549	520.2319	475.2134	375.8459	348.792
418.5	598.2111	578.4176	517.5102	477.7754	365.2042	577.7047	521.4949	476.2319	376.8247	349.9742
419	601.3025	581.3669	518.176	478.7656	366.0203	579.0356	522.5087	477.7961	377.9154	350.8261
419.5	603.9782	584.0249	518.668	479.226	366.7072	580.2015	523.8058	479.5758	378.9716	351.6032
420	605.741	585.9862	519.4914	479.9685	367.3083	581.4639	524.5422	481.502	379.8974	352.6511
420.5	607.2386	587.9908	520.4643	481.3877	367.847	583.0088	525.4788	483.2137	380.5658	353.7242
421	608.1423	589.7812	522.0422	482.4932	368.4006	584.6404	526.1198	484.6597	381.2242	354.593
421.5	608.7138	591.4224	523.9006	483.6402	368.9789	586.7845	526.7649	485.5478	382.2478	355.5004
422	608.7399	592.786	526.1484	484.5796	369.6952	589.1712	527.2852	485.9987	383.1517	356.265
422.5	607.7637	593.5995	528.9739	485.2826	370.4079	591.5268	528.1459	486.2338	384.1488	356.8685
423	606.7718	594.2989	531.3137	486.049	371.3533	593.4852	529.1306	486.3882	384.8541	357.4419
423.5	606.2263	595.4948	532.9021	486.4659	372.0546	594.4261	530.0379	486.6853	385.4943	357.2451
424	606.2529	596.3696	533.5984	486.3823	372.5504	595.3685	531.2694	486.9492	385.7048	356.9007
424.5	605.7828	596.6134	533.4511	486.2035	373.0619	595.6644	532.1786	487.3938	385.3768	356.6731
425	606.2881	596.7445	532.9109	486.1989	373.5371	595.5738	533.1672	487.7365	384.7062	356.5211
425.5	606.9596	596.9152	532.4319	486.6513	373.6619	595.0634	533.3072	487.9183	383.7221	356.3624
426	607.955	597.2732	531.7637	487.0542	374.1325	594.862	533.2044	487.5305	382.8458	356.4015
426.5	609.0381	597.6512	531.1774	487.3324	374.3408	595.2181	532.3863	486.8006	381.9924	356.6318
427	609.3331	597.8897	531.3326	487.4134	374.4325	595.6624	531.148	486.087	381.9491	356.8787
427.5	609.4269	597.7432	531.2896	487.461	374.4693	596.4933	530.0093	484.9053	382.567	357.4713
428	609.6746	597.7779	530.959	487.3562	374.0854	597.2127	528.7009	484.0937	383.7352	357.6141
428.5	610.3199	597.7011	529.9402	487.2297	373.5267	598.3681	527.3839	483.3693	384.8552	357.9258
429	610.2045	597.1105	528.5564	486.9346	372.7779	599.0233	526.6303	483.2256	385.2995	358.2778
429.5	610.2686	596.2145	527.2414	486.274	371.9835	599.0071	526.4627	482.9604	385.6127	358.2344
430	609.5644	594.9154	526.1619	486.2724	370.4773	597.7928	525.9475	482.8961	385.6092	357.9241

430.5	608.4989	593.5274	524.8636	486.1027	369.3935	595.7174	526.0051	482.6999	385.0702	357.3895
431	607.5072	592.1911	523.7411	485.6542	368.6386	593.8986	526.2819	482.1194	384.0506	356.6974
431.5	605.7293	591.088	523.3604	484.6019	367.9535	591.484	526.0098	481.6793	383.1107	355.9127
432	603.7616	589.6006	523.3833	482.8405	367.3675	589.2384	525.9501	480.2055	382.4469	355.0249
432.5	602.0765	588.4588	523.2378	480.5156	366.9788	587.6291	525.5455	478.8378	382.1917	353.884
433	600.5856	587.2272	521.9532	478.1217	366.9985	586.8311	524.6479	477.0703	382.2405	353.1267
433.5	598.8969	585.623	519.9522	475.831	367.1439	586.3861	523.7755	475.5915	381.5076	352.8445
434	597.4059	584.2112	517.9463	473.2702	367.6494	585.7302	522.6707	473.8474	380.6085	352.7706
434.5	595.278	582.4411	515.9975	471.1868	367.6379	584.7797	520.9306	472.0569	379.6985	352.8782
435	593.0341	580.4819	513.5536	469.3722	367.2164	583.2487	519.166	470.4597	378.5979	353.0956
435.5	590.9743	578.5335	511.3214	467.6736	366.7498	581.6459	517.647	468.8338	377.1919	353.2749
436	588.3355	576.7124	509.6126	465.816	365.6714	579.0286	515.4715	467.5965	376.2589	353.6578
436.5	585.4	574.5868	508.6002	463.3896	363.8665	575.794	513.2129	465.5688	375.6018	353.6855
437	582.691	572.776	507.846	460.7295	361.4181	572.8712	510.8471	463.6077	374.8763	353.0158
437.5	579.982	570.9243	506.5635	458.2405	358.9571	570.3	508.3417	461.2238	374.4703	352.0888
438	577.0968	568.7137	504.4535	456.0797	356.8109	568.2339	505.8501	458.9285	373.7206	351.2424
438.5	573.968	566.5405	502.0917	454.0937	355.2631	565.4426	502.6745	456.4338	372.6125	350.0669
439	570.3545	563.9404	499.3669	452.3427	354.116	562.5459	499.3661	453.7557	371.5433	348.5793
439.5	566.6726	560.9943	496.0536	451.0999	353.1425	559.4723	496.2503	451.0101	370.264	346.8847
440	563.1226	557.6941	492.6012	450.2103	352.5185	556.2516	493.7459	448.3086	368.4956	344.8438
440.5	559.4256	553.8913	489.0135	449.2115	351.8016	552.4205	491.2731	445.8491	367.0041	343.0242
441	555.421	549.853	485.756	447.6841	350.9645	548.1219	488.7236	443.4523	365.829	341.294
441.5	551.6808	545.7025	483.0757	445.8403	349.6506	543.9946	486.3054	441.3727	364.482	339.0938
442	548.666	541.5706	480.9454	443.6651	347.8816	540.3979	484.2732	439.3218	363.0546	337.0936
442.5	545.768	537.5432	478.7284	441.279	345.7963	537.88	482.5277	437.5597	361.5238	335.7703
443	542.4202	533.5032	476.5876	438.9279	343.4337	534.8826	480.0286	435.9129	359.8409	334.8654
443.5	538.8913	529.7987	474.0359	436.5858	341.0842	531.7325	476.9727	434.3122	358.3018	334.4552
444	535.6238	525.994	470.8759	434.5996	338.9581	528.8669	474.0202	432.54	356.9645	334.324
444.5	532.6506	522.157	467.4076	432.8846	336.7824	526.1202	471.1646	430.6387	355.5935	334.4491
445	529.85	518.3016	463.6475	431.324	334.5634	522.9905	468.1092	428.463	354.4244	334.9647
445.5	526.4279	514.7023	459.5624	429.5681	332.7594	519.5678	464.6537	426.1639	353.7263	335.5638
446	522.7827	511.1075	455.5025	427.8412	331.1956	516.0131	460.8484	423.8488	353.0365	335.366
446.5	519.3331	507.6066	451.5549	425.9192	329.7412	512.8193	457.1096	421.4645	352.0754	334.8366

447	515.772	504.2202	448.0437	423.6732	328.4824	510.0082	453.6307	419.2339	350.8148	334.3106
447.5	511.7348	500.7516	445.201	421.3938	327.0138	507.3059	449.968	417.0674	349.6636	333.4527
448	507.6466	497.8788	442.3573	418.7344	325.4462	504.4948	445.6199	415.0338	348.7595	332.6566
448.5	503.6593	494.7757	439.5709	415.6765	324.0104	501.7812	441.7313	413.2116	348.11	331.7199
449	499.8639	491.3468	436.9962	412.5419	322.1904	498.9174	438.4226	411.4291	347.5812	330.901
449.5	496.6186	488.1157	434.4927	409.7376	319.9162	495.9445	435.7128	409.5696	346.9313	330.4115
450	492.9797	485.0527	431.9864	407.022	318.0172	493.1735	433.4515	407.5478	346.3608	329.9978
450.5	489.3645	482.2362	429.9969	404.5798	316.4057	490.0969	431.4918	405.1437	345.4907	329.0628
451	486.0205	479.6854	427.5509	402.2476	315.0605	487.0878	429.753	402.6702	344.2091	327.6811
451.5	482.82	477.2972	425.4333	399.6477	314.0461	483.9134	428.546	400.2666	342.2958	326.5766
452	479.7068	474.5972	423.2579	397.7093	313.0321	480.9306	427.7115	397.6979	340.314	325.2894
452.5	477.2184	472.4018	420.7969	395.8116	312.2755	477.6627	426.1878	395.0363	338.4554	324.2694
453	474.7292	470.0877	418.0271	393.5676	311.5251	474.2289	424.4592	392.7155	337.1665	323.3686
453.5	472.5835	467.1679	414.8922	390.8439	310.3712	470.482	422.433	390.8876	336.3355	322.6662
454	470.8342	464.1929	411.5438	388.2621	308.6105	466.6553	420.1562	389.2579	335.9495	322.0317
454.5	468.3576	460.7519	407.6722	385.8233	307.0589	463.4751	417.5507	387.7645	335.7773	321.4087
455	465.2698	457.114	404.1019	383.4523	305.4277	460.3485	414.7842	386.1828	335.4656	320.6757
455.5	461.9149	453.4872	400.4546	381.276	303.7177	457.492	411.6799	384.4514	335.0198	319.4082
456	457.778	449.8345	397.3853	378.5966	301.7847	454.6036	408.6186	382.8755	333.8526	318.3845
456.5	453.1854	445.6852	394.644	376.4074	299.9014	452.3326	405.8595	380.9399	332.3814	317.4065
457	449.1301	441.6681	391.9488	375.0007	298.8074	450.1908	402.6886	378.7817	330.7883	316.5352
457.5	445.0908	438.0335	389.2799	373.6378	297.8232	448.1195	399.3885	376.2531	329.4471	316.1419
458	441.4118	434.5834	386.984	371.9583	296.4315	445.685	395.8502	373.7833	328.132	316.3764
458.5	438.7475	431.3319	385.0329	370.1753	294.8414	443.1543	392.5147	371.2655	327.376	316.5225
459	436.3866	428.2814	383.0399	368.5375	293.3685	441.0193	389.4616	369.2548	326.6035	316.6847
459.5	433.7982	425.3435	380.8689	366.832	291.643	439.0447	386.8633	367.5493	325.5224	316.7007
460	431.5841	422.3614	378.5817	365.2897	289.892	436.913	384.1002	365.9077	324.4109	316.0211
460.5	428.9166	419.4753	376.1665	363.2387	287.5623	434.363	381.5696	364.1485	323.1916	314.9866
461	425.8581	416.0262	373.6092	360.8328	284.9075	431.9035	379.6791	361.9581	321.8209	313.4024
461.5	422.9762	412.1121	371.0705	358.9439	282.7327	428.9701	377.6905	359.667	320.7369	311.4479
462	419.7308	407.9842	367.7467	357.2853	280.6751	425.6108	375.5917	356.851	319.9518	309.6397
462.5	416.3033	404.3161	364.1403	355.4289	278.5314	421.7395	373.1204	354.0373	319.1296	308.3307
463	413.3607	400.5198	360.4297	353.2697	276.9458	417.5243	370.4429	351.0192	319.059	307.2308

463.5	410.3449	397.1415	356.9594	350.917	276.0331	413.1288	367.7622	348.5953	319.1799	306.218
464	406.8419	394.1554	353.4261	348.4604	275.3181	409.2661	365.2071	346.3998	319.2057	305.6618
464.5	403.5896	391.1943	350.3584	345.9615	275.1689	405.6207	362.4653	344.8864	318.7479	305.4193
465	400.1428	388.6889	347.0947	343.3946	274.85	402.0539	359.6314	343.4835	317.8322	305.3799
465.5	396.6631	386.1261	344.1363	340.2475	274.1858	399.1878	357.3089	341.7967	316.1686	305.1121
466	393.2671	383.1938	341.9238	337.1788	273.2566	396.516	355.197	340.2511	314.5678	304.3674
466.5	390.0409	379.6925	339.6684	334.5929	271.9043	393.8463	353.1413	338.31	312.9506	303.4802
467	386.7505	376.6431	337.309	331.8849	269.9657	391.0992	350.7278	336.4853	311.061	302.421
467.5	384.2244	373.6955	334.5599	329.1608	267.8428	388.1431	347.8977	334.371	309.3363	301.4504
468	381.9186	371.0091	331.7333	326.0023	265.718	384.7203	344.8228	332.6102	307.9707	300.0919
468.5	379.1224	368.6006	328.6336	323.1817	263.1375	381.6522	341.613	330.5941	306.9664	298.7913
469	376.2368	365.8545	325.7679	320.6512	260.934	378.5366	338.4246	329.1035	305.4129	297.8653
469.5	373.1919	363.1574	322.7444	318.7153	259.0477	375.0663	335.1224	327.8161	303.9072	297.0782
470	370.1547	360.3208	319.818	317.0981	257.3877	371.7919	332.1778	326.314	301.9431	296.0897
470.5	367.2677	357.4121	317.1808	315.5533	255.7123	368.6831	329.9302	324.8061	300.2065	294.9319
471	364.3919	353.8919	314.9582	314.794	254.1226	365.4429	328.1556	322.6005	298.7988	293.8414
471.5	361.2738	350.3106	312.9391	314.1214	252.8211	362.2759	326.6965	320.231	297.6884	292.8947
472	358.8302	346.9042	311.219	313.2657	251.9011	359.1936	325.259	317.6404	296.7767	292.5544
472.5	357.1463	343.7057	309.5073	311.4939	251.1586	356.0738	323.7282	315.4102	296.2654	292.1605
473	355.3123	340.7087	307.5273	309.2682	250.1737	353.5706	321.7163	313.1768	296.1897	291.9955
473.5	353.322	337.5406	305.3369	306.9286	249.4037	351.6185	319.7387	311.2431	295.9695	292.1351
474	350.9976	334.5055	302.8894	304.4821	248.6297	349.5737	317.2501	309.8896	295.833	292.2349
474.5	348.5278	331.6551	300.3214	302.2909	247.8186	347.6611	314.7161	308.6509	295.1764	291.9696
475	346.3876	329.6898	297.3833	299.7497	246.6423	346.2995	312.4561	307.8994	294.2556	291.3182
475.5	344.2167	327.9318	294.2829	297.6526	245.4329	345.0073	310.0475	307.222	293.2547	290.254
476	341.7235	326.155	290.968	296.0884	244.1241	343.4323	307.8267	306.6258	292.4222	288.7678
476.5	339.1814	324.4643	288.1858	294.6732	243.0939	341.6785	305.7382	306.0446	291.8095	287.3402
477	337.4186	322.7424	285.9785	293.2465	242.0659	339.5424	303.8274	305.7063	291.2016	285.5304
477.5	335.6133	320.8174	284.4006	291.7679	240.8145	337.5589	301.7018	305.4141	290.9288	284.0015
478	333.9465	318.331	283.2091	290.532	239.6085	336.2446	299.7765	305.3752	290.6228	282.7992
478.5	331.8101	315.156	282.3816	289.0967	238.3964	334.8042	297.5602	305.3527	290.7839	281.8032
479	329.446	311.5527	281.81	287.9568	237.2298	333.211	295.5781	304.9974	290.915	280.946
479.5	327.4085	308.3777	281.2416	286.6016	235.8662	331.7997	293.9575	304.8202	290.8968	280.7297

480	325.2191	305.5032	280.11	285.3052	234.4419	330.4771	292.6002	304.804	290.8037	281.0336
480.5	323.1563	302.8163	278.1309	284.1733	233.0135	328.89	291.559	304.7057	290.5847	281.3981
481	321.0449	300.5502	275.9795	282.8538	231.9418	327.3122	290.6853	304.2835	290.6101	282.0309
481.5	319.2153	298.8345	273.7127	281.8157	231.059	325.2064	289.9891	304.1182	290.3622	282.2394
482	317.5292	297.66	271.5905	280.9656	230.3001	322.9885	289.2444	303.9452	289.9837	282.4179
482.5	316.1536	296.7859	269.6197	280.5506	229.6527	321.3024	288.3489	304.5546	289.2667	282.073
483	314.3607	295.782	267.7863	280.0064	229.172	319.7106	287.2494	305.4624	288.7745	281.3246
483.5	312.7131	294.8194	266.2199	279.7858	229.0706	317.8668	285.7467	306.1197	288.3348	280.2278
484	311.5716	294.0916	265.3511	279.5064	229.114	316.0726	283.7901	306.9661	287.9322	279.3857
484.5	310.3715	293.4875	264.575	279.0991	228.9258	314.2576	281.6943	307.8904	287.4685	279.3367
485	309.4384	292.5411	263.6571	277.9587	228.512	312.4462	279.8139	309.0237	286.9106	279.5314
485.5	308.7192	291.3976	262.6159	276.18	228.1039	311.0101	278.1638	309.905	286.8815	280.2169
486	308.0992	289.6857	261.6102	274.0286	227.7645	309.2066	276.8382	307.9707	286.7962	281.114
486.5	307.5891	287.3881	260.7001	272.2514	227.2848	307.321	275.9769	306.9664	286.7455	282.0765
487	307.2446	285.2654	260.0199	270.8227	226.4524	305.6676	275.2656	305.4129	286.4822	282.5134
487.5	306.1968	283.0518	259.0545	269.4657	225.2808	304.5003	275.04	303.9072	286.1223	282.4843
488	305.2389	281.2002	257.567	268.5341	224.0824	303.4693	274.5895	301.9431	285.4451	281.8584
488.5	304.2814	279.4616	256.2305	268.1652	223.2891	302.8272	273.9433	300.2065	284.7273	280.8804
489	302.9165	278.2452	254.9116	268.3204	222.4466	302.026	273.1725	298.7988	283.7614	279.9386
489.5	301.4915	277.1048	253.6167	268.1435	221.4372	301.1148	272.4838	297.6884	282.6876	279.1468
490	300.1761	276.6377	252.0877	267.6565	220.3323	300.3051	271.7854	296.7767	281.8932	278.6839
490.5	298.8039	276.073	250.5849	266.8199	219.3633	299.2164	270.9877	296.2654	281.5339	278.7322
491	297.6643	274.7906	249.2974	265.9464	218.7767	298.1459	270.3566	296.1897	281.5101	278.8718
491.5	296.6007	273.7833	248.4975	265.051	218.4782	296.804	269.6541	295.9695	281.8377	278.9696
492	295.2175	272.7603	248.0931	264.2362	218.2883	295.7054	269.1405	295.833	282.4879	279.0126
492.5	294.1267	271.8394	247.4741	263.5058	218.222	294.5305	268.2416	295.1764	282.8324	279.1724
493	293.2654	270.732	246.7695	263.1778	218.5137	293.5749	267.3789	294.2556	283.2052	279.2929
493.5	292.4979	269.8418	246.119	263.2176	219.0971	292.708	266.3445	293.2547	283.319	279.438
494	291.8804	268.9465	245.6061	263.2769	219.7199	292.1076	265.384	292.4222	283.4548	279.404
494.5	291.5616	268.6896	244.7848	263.2521	220.0208	291.5346	264.4388	291.8095	283.571	279.3254
495	291.4937	268.678	243.7691	262.9836	219.8472	291.0098	263.5087	291.2016	283.6159	279.5535
495.5	291.8701	267.8244	242.6547	262.4913	219.5701	290.6316	262.6307	290.9288	283.766	279.8163
496	292.1129	266.8962	241.4592	261.5412	219.1098	290.2204	262.0617	290.6228	284.004	280.1786

496.5	292.1492	265.5531	240.8687	260.8044	218.7528	288.543	261.9704	290.7839	285.3783	279.1293
497	292.3611	263.9441	240.7329	260.2648	218.486	285.2819	261.9697	290.915	287.2727	276.938
497.5	292.1507	262.2899	240.375	259.5657	218.0403	282.7393	261.7919	290.8968	288.2347	275.6253
498	291.6889	261.0706	239.7362	258.6139	217.3826	282.2518	260.7935	290.8037	287.5356	277.2059
498.5	290.5854	260.7091	238.6146	257.1034	216.2346	285.4224	258.9039	290.5847	284.433	282.5549
499	289.2181	261.9007	236.7295	255.1474	214.3234	292.2755	255.7795	290.6101	278.3401	292.2605
499.5	287.3277	264.8831	234.0775	252.8226	211.3867	303.513	251.4392	290.3622	269.0543	306.5057
500	285.5452	268.8526	230.745	249.9631	207.7525	318.7951	245.8084	289.9837	257.2312	325.3278

Table 9. Data from Fig. 6 B

Sample	Area	Percent (%)
**70 °C-100 mM**	31849.66	46.641
**70 °C-50 mM**	18609.41	27.252
**70 °C-25 mM**	8552.125	12.524
**70 °C-10 mM**	9275.397	13.583

Table 10. Data from Fig. 7 B

Sample	Area	Percent (%)
**50 °C-10 mM**	23630.9	18.892
**50 °C-25 mM**	33497.31	26.78
**50 °C-50 mM**	36175.36	28.921
**50 °C-100 mM**	31781.77	25.408

Table 11. Data from Fig. 7 C

**0 h**	**0 mM**	**10 mM**	**25 mM**	**50 mM**	**100 mM**
**50 °C**	610.31	597.88	533.59	487.41	374.46
**70 °C**	599.02	533.3	487.91	385.7	357.44
**3 h**					
**50 °C**	567	548.15	552.11	463.4	449.72
**70 °C**	548.48	524.5	460.33	382.77	411.17
**48 h**					
**50 °C**	567.05	534.02	515.26	458.88	427.95
**70 °C**	543.54	501.2	476.89	418.36	439.67

Table 12. Data from Fig. 8 A

**0 h**	0 mM	50 mM	100 mM
**50 °C**	773.68	568.52	588.81
**70 °C**	967.11	562.93	603.28
**3 h**			
**50 °C**	672.77	530.96	506.21
**70 °C**	840.96	591.94	567.33
**48 h**			
**50 °C**	614.8	548.9	523.19
**70 °C**	720.19	611.97	548.1

Table 13. Data from Fig. 8 B

Sample / Time (sec)	Den	Art 0.5- 25 °C	Art 0.5- 50 °C	Art 0.5- 60 °C	Art 0.5- 70 °C	Art 0.5- 80 °C
**0**	0.521	0.494	0.475	0.554	0.558	0.542
**10**	0.533	0.507	0.49	0.565	0.571	0.557
**20**	0.537	0.512	0.495	0.569	0.575	0.562
**30**	0.539	0.515	0.499	0.57	0.576	0.564
**40**	0.542	0.517	0.5	0.574	0.579	0.565
**50**	0.541	0.516	0.499	0.572	0.577	0.566
**60**	0.54	0.517	0.5	0.573	0.577	0.567
**70**	0.54	0.518	0.501	0.574	0.577	0.568
**80**	0.542	0.52	0.5	0.575	0.582	0.569
**90**	0.543	0.519	0.5	0.575	0.583	0.569
**100**	0.542	0.517	0.501	0.575	0.582	0.568
**110**	0.542	0.518	0.501	0.576	0.58	0.568
**120**	0.544	0.517	0.502	0.578	0.582	0.568

Table 14. Data from Fig. 9 A

Sample / Time (sec)	Den	Art 1- 25 °C	Art 1- 50 °C	Art 1- 60 °C	Art 1- 70 °C	Art 1- 80 °C
**0**	0.521	0.432	0.423	0.498	0.503	0.504
**10**	0.533	0.446	0.433	0.509	0.517	0.519
**20**	0.537	0.45	0.437	0.514	0.522	0.524
**30**	0.539	0.453	0.439	0.516	0.525	0.526
**40**	0.542	0.454	0.44	0.517	0.526	0.527
**50**	0.541	0.455	0.44	0.518	0.526	0.529
**60**	0.54	0.455	0.44	0.519	0.527	0.529
**70**	0.54	0.455	0.441	0.52	0.528	0.528
**80**	0.542	0.456	0.442	0.521	0.527	0.532
**90**	0.543	0.456	0.442	0.521	0.528	0.529
**100**	0.542	0.456	0.442	0.521	0.529	0.533
**110**	0.542	0.457	0.442	0.52	0.53	0.53
**120**	0.544	0.457	0.443	0.521	0.528	0.53

Table 15. Data from Fig. 9 B

Sample / Time (sec)	Den	Art 0.5-H2O2 0	Art 0.5-H2O2 25	Art 0.5-H2O2 50	Art 0.5 -H2O2 100
**0**	0.638	0.638	0.514	0.55	0.628
**10**	0.654	0.647	0.529	0.564	0.641
**20**	0.662	0.653	0.534	0.57	0.645
**30**	0.665	0.654	0.535	0.568	0.647
**40**	0.666	0.654	0.539	0.574	0.65
**50**	0.667	0.655	0.537	0.57	0.65
**60**	0.667	0.655	0.539	0.575	0.651
**70**	0.668	0.657	0.539	0.571	0.652
**80**	0.669	0.659	0.539	0.573	0.652
**90**	0.67	0.659	0.54	0.574	0.653
**100**	0.67	0.66	0.54	0.573	0.654
**110**	0.671	0.659	0.541	0.575	0.655
**120**	0.67	0.657	0.542	0.575	0.654

Table 16. Data from Fig. 9 C

Sample / Time (sec)	Den	Art 1-H2O2 0	Art 1-H2O2 25	Art 1 -H2O2 50	Art 1 -H2O2 100
**0**	0.638	0.534	0.455	0.586	0.628
**10**	0.654	0.54	0.466	0.599	0.641
**20**	0.662	0.546	0.471	0.602	0.645
**30**	0.665	0.546	0.475	0.606	0.647
**40**	0.666	0.551	0.474	0.608	0.65
**50**	0.667	0.546	0.477	0.609	0.65
**60**	0.667	0.554	0.475	0.61	0.651
**70**	0.668	0.552	0.473	0.61	0.652
**80**	0.669	0.552	0.474	0.61	0.652
**90**	0.67	0.555	0.477	0.612	0.653
**100**	0.67	0.551	0.478	0.611	0.654
**110**	0.671	0.554	0.479	0.61	0.655
**120**	0.67	0.552	0.481	0.61	0.654

Table 17. Data from Fig. 9 D

Sample / Time (sec)	Den	Art 25-H2O2 25	Art 50-H2O2 25	Art 70-H2O2 25	Art 25-H2O2 50	Art 50-H2O2 50	Art 70-H2O2 50	Art 25-H2O2 100	Art 50-H2O2 100	Art 70-H2O2 100
**0**	0.58	0.5	0.423	0.452	0.499	0.456	0.53	0.395	0.392	0.445
**10**	0.594	0.522	0.441	0.475	0.51	0.471	0.551	0.411	0.407	0.459
**20**	0.598	0.521	0.449	0.481	0.515	0.477	0.561	0.419	0.416	0.466
**30**	0.603	0.522	0.453	0.487	0.519	0.477	0.564	0.422	0.42	0.469
**40**	0.605	0.527	0.455	0.488	0.525	0.482	0.574	0.424	0.42	0.471
**50**	0.603	0.529	0.456	0.491	0.531	0.484	0.56	0.425	0.42	0.472
**60**	0.606	0.531	0.457	0.492	0.529	0.482	0.563	0.426	0.42	0.474
**70**	0.611	0.529	0.459	0.493	0.529	0.484	0.566	0.427	0.421	0.473
**80**	0.613	0.527	0.458	0.493	0.527	0.486	0.566	0.428	0.422	0.478
**90**	0.615	0.527	0.46	0.494	0.529	0.489	0.566	0.428	0.423	0.478
**100**	0.614	0.52	0.46	0.495	0.533	0.49	0.568	0.428	0.423	0.477
**110**	0.614	0.525	0.461	0.494	0.529	0.49	0.565	0.429	0.423	0.478
**120**	0.614	0.517	0.462	0.495	0.53	0.489	0.567	0.429	0.423	0.477

Table 18. Data from Fig. 10
