# Supplementary figures and images for: Stress-dependent conformational changes of artemin: Effects of heat and oxidant
Source: PLoS One. 2020 Nov 16;15(11):e0242206. doi: 10.1371/journal.pone.0242206 (PMC7668597; doi:10.1371/journal.pone.0242206)

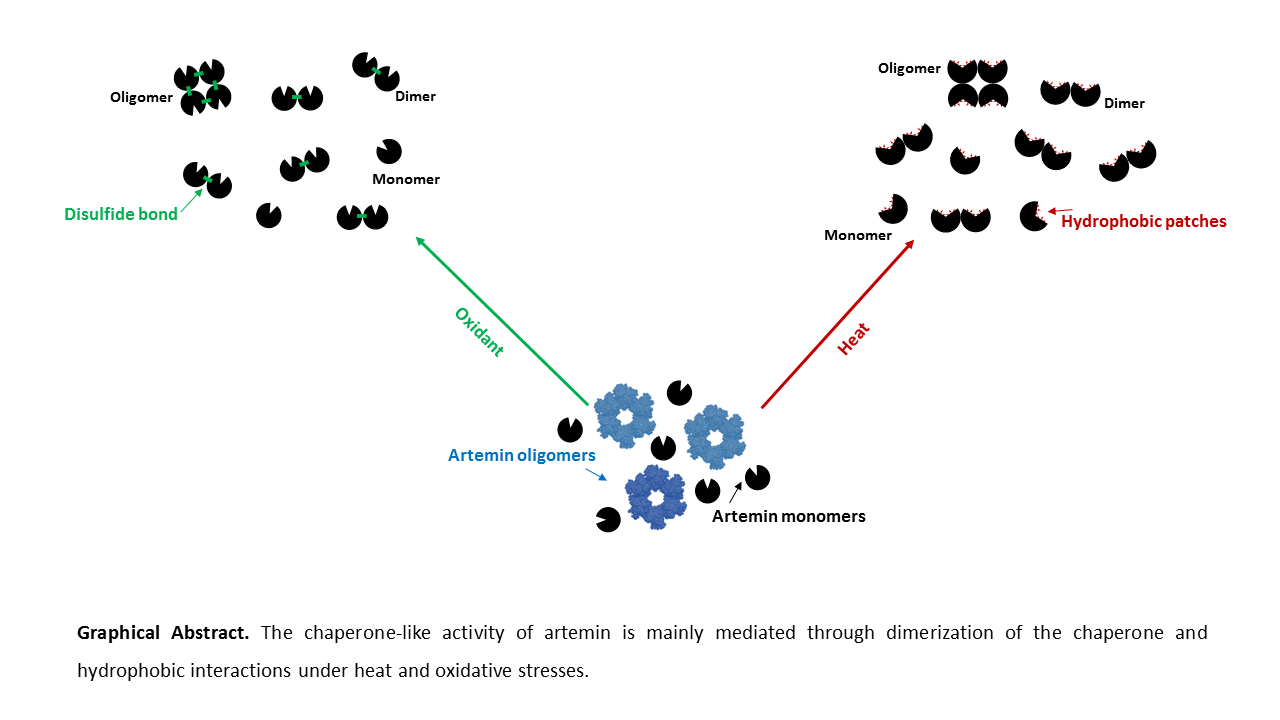

Supplement: S1 Graphical abstract — (TIF) [file pone.0242206.s003.tif]

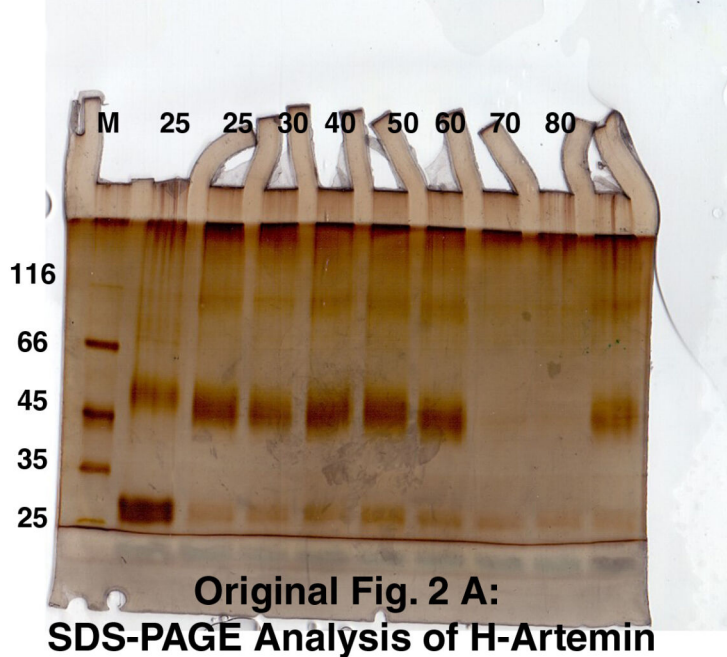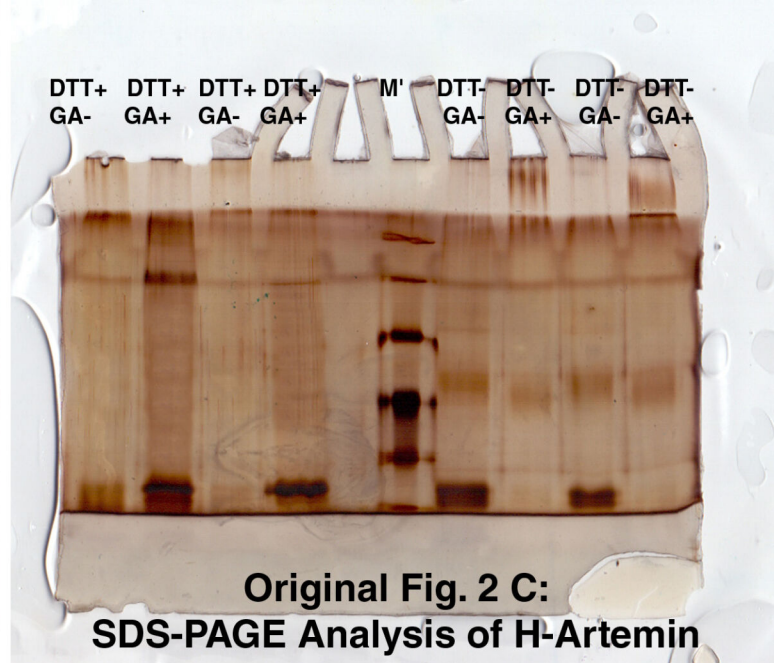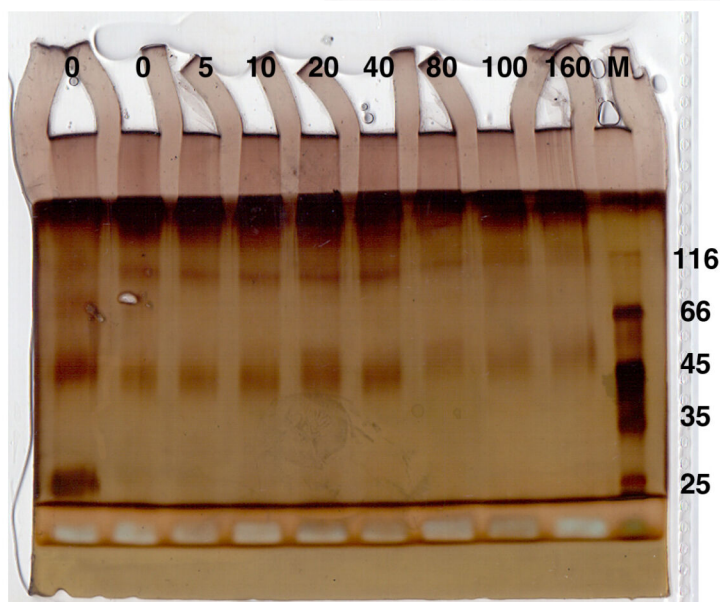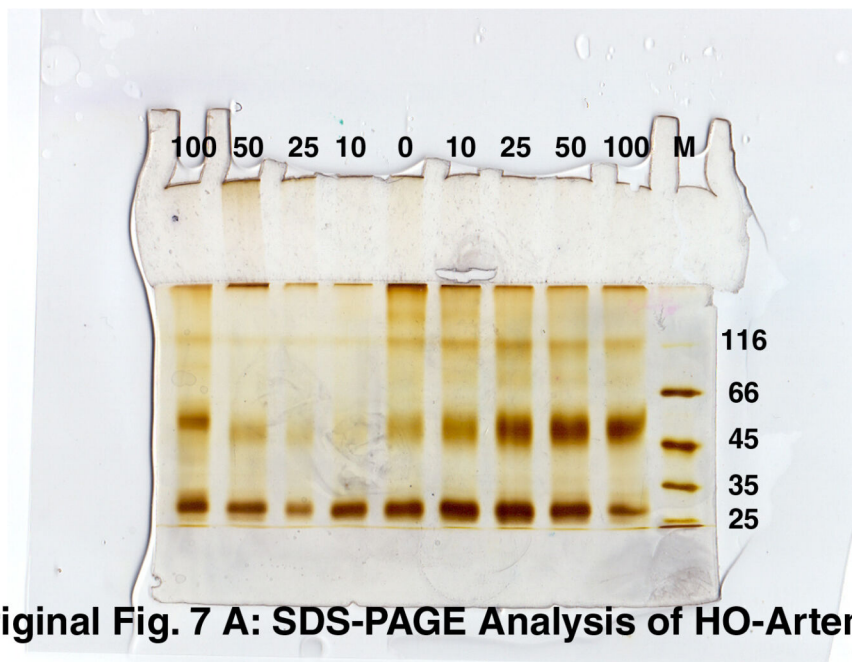

Supplement: S1 Raw images — (PDF) [file pone.0242206.s004.pdf]

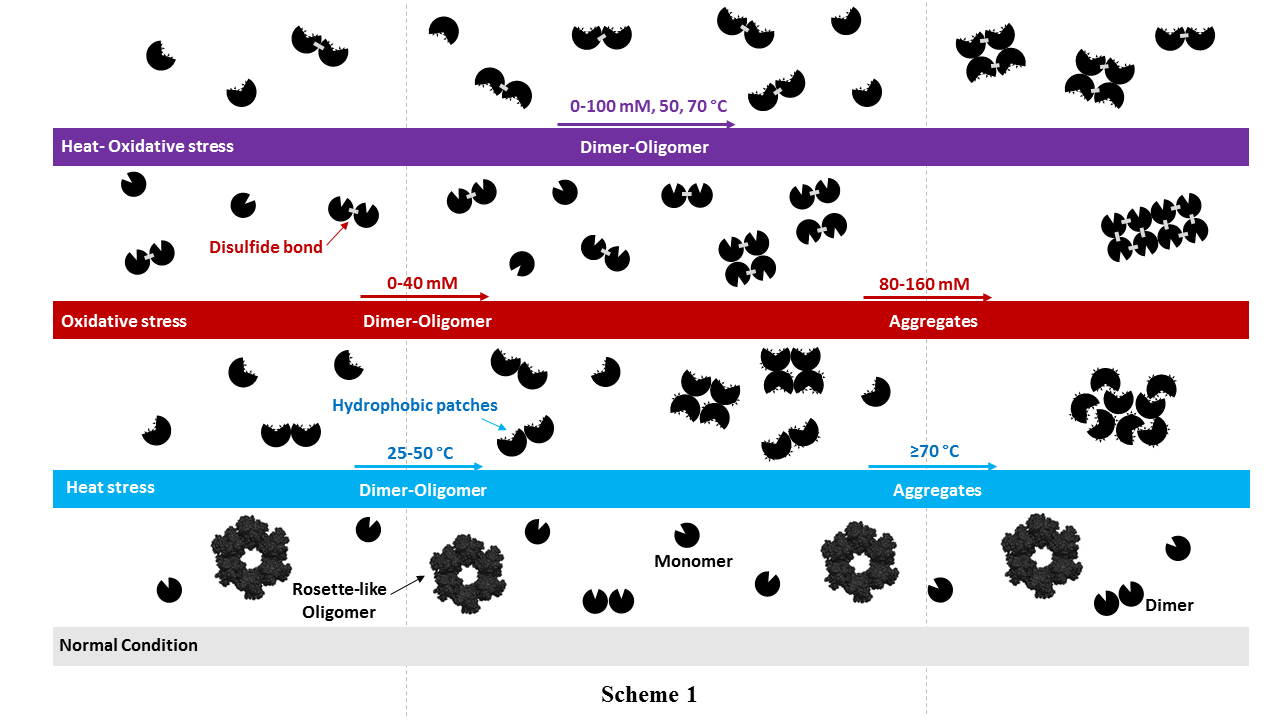

Supplement: S1 Scheme — (TIF) [file pone.0242206.s005.tif]
