# Supplementary material for: Stress-dependent conformational changes of artemin: Effects of heat and oxidant
Source: PLoS One. 2020 Nov 16;15(11):e0242206. doi: 10.1371/journal.pone.0242206 (PMC7668597; doi:10.1371/journal.pone.0242206)
Supplement: Attachment — Submitted filename: Response to editor.docx [file pone.0242206.s007.docx]

**Dear Editors-in-Chief,**

We are grateful for your consideration of this manuscript (Manuscript ID: PONE-D-20-06479). All the comments we received on this study have been taken into account in improving the quality of the article, and we present our reply to each of them separately. All corrections and modifications are highlighted in red in the revised version of the manuscript.

1. The numbers identifying the affiliations of authors were checked and corrected (Page 1).
2. We re-read the whole manuscript and corrected the grammatical errors and typing mistakes as much as possible, e.g.: Page 2; Lines 4, 5, 6, 7, 10, 14, Page 3; Line 11, Page 4; Lines 1, 3, 20, 21, Page 6; Lines 5, 6, 8, Page 9; Lines 9, 10, Page 10; Lines 1, 9, 16, Page 11; Lines 3, 4, 6, 21, Page 12; Line 11, Page 16; Lines 10, 16, 17, Page 17; Lines 1, 3, 14, 19, 20, Page 18; Lines 9, 10, 13, 14, Page 19; Lines , 18, 20, Page 20; Lines 3, 4, 14, Page 21; Lines 5, 15, 18, 20, 22, 23, Page 22; Lines 1, 3, 6, 9, 14, 21, Page 23; Lines 1, 9, 10, Page 24; Lines 17, 18, 22, Page 25; Line 18, Page 26; Line 9, Page 27; Lines 5, 19, 20, 23.
3. Regarding the conclusion section, as the respectful reviewer recommended us to add some related information about the physiology of *Artemia* cysts in the conclusion section, and accordingly the conclusion was too lengthy. As the dear editor suggested, we removed the first part of the conclusion and added that to the discussion (Page 28; Lines 3-18), and shortened conclusion as much as possible (Page 28; Lines 21-22, Page 29; Lines 1-6).
4. We corrected the scheme 1 and graphical abstract as the respectful reviewer suggested.

Again, we appreciate you taking the time to offer us your comments and insights related to the paper. We hope that these changes to the manuscript will facilitate the decision to publish this study in your journal. In any case, we are open to consideration of any further comment on our manuscript.

Sincerely,

Reza H Sajedi, PhD

Professor of Biochemistry

Department of Biochemistry,

Faculty of Biological Sciences,

Tarbiat Modares University, Tehran, Iran

E.mail: [sajedi_r@modares.ac.ir](mailto:sajedi_r@modares.ac.ir)
